# Mitochondrial genomes reveal mid-Pleistocene population divergence, and post-glacial expansion, in Australasian snapper (*Chrysophrys auratus*)

**DOI:** 10.1038/s41437-022-00579-1

**Published:** 2022-12-03

**Authors:** Tom Oosting, Lourdes Martínez-García, Giada Ferrari, Alexander J. F. Verry, Lachie Scarsbrook, Nicolas J. Rawlence, Maren Wellenreuther, Bastiaan Star, Peter A. Ritchie

**Affiliations:** 1 Victoria University of Wellington, School of Biological Sciences, Wellington, New Zealand; 2 University of Oslo, Centre for Ecological and Evolutionary Synthesis (CEES), Oslo, Norway; 3 Université de Toulouse, Centre for Anthropobiology and Genomics, Toulouse, France; 4 University of Oxford, School of Archaeology, Palaeogenomics and Bio-archaeology Research Network, Oxford, England; 5 University of Otago, Department of Zoology, Dunedin, New Zealand; 6 The New Zealand Institute for Plant & Food Research Ltd & University of Auckland, Auckland, New Zealand

**Keywords:** Population genetics, Phylogenetics, Evolutionary genetics, Population genetics, Evolutionary biology

## Abstract

Glacial cycles play important roles in determining the phylogeographic structure of terrestrial species, however, relatively little is known about their impacts on the distribution of marine biota. This study utilised modern (*n* = 350) and ancient (*n* = 26) mitochondrial genomes from Australasian snapper (*Chrysophrys auratus*) sampled in New Zealand to assess their demographic and phylogeographic history. We also tested for changes in genetic diversity using the up to 750-year-old mitochondrial genomes from pre-European archaeological sites to assess the potential impacts of human exploitation. Nucleotide diversity and haplotype diversity was high (π = 0.005, *h* *=* 0.972). There was no significant change in nucleotide diversity over the last 750 years (*p* = 0.343), with no detectable loss of diversity as a result of indigenous and industrial-scale fishing activity. While there was no evidence for contemporary population structure (AMOVA, *p* = 0.764), phylogeographic analyses identified two distinct mitochondrial clades that diverged approximately 650,000 years ago during the mid-Pleistocene, suggesting the species experienced barriers to gene flow when sea levels dropped over 120 m during previous glacial maxima. An exponential population increase was also observed around 8000 years ago consistent with a post-glacial expansion, which was likely facilitated by increased ocean temperatures and rising sea levels. This study demonstrates that glacial cycles likely played an important role in the demographic history of *C. auratus* and adds to our growing understanding of how dynamic climatic changes have influenced the evolution of coastal marine species.

## Introduction

Phylogeographic studies have provided key insights into past demographic changes such as population expansion, contraction and fragmentation. Geological processes such as continental drift, mountain formation, climatic changes, shifting sea currents, and Pleistocene glacial-interglacial cycles have been shown to play important roles in the evolution and phylogeographic structuring of biodiversity (Teske et al. 2011; Wallis and Trewick 2009). Glacial cycles are often considered to have had regular and significant impacts on the phylogeographic structure and population demographics of many species (Hewitt 2000). Long-term extrinsic barriers to gene flow typically produce deep divergences in gene trees, which persist for long periods of time in non-recombining genetic markers, such as the mitochondrial genome (Harpending et al. 1998). In terrestrial ecosystems, the formation of glaciers and ice sheets has led to widespread habitat loss and disruption to gene flow among populations, which resulted in phylogeographic structure that is often similar among a range of taxa (Wallis et al. 2016). For example, common phylogeographic patterns observed between terrestrial species in North America were attributed to isolation in refugia during the Last Glacial Maximum (LGM) (Shafer et al. 2010). Similarly, red deer in Europe were observed to have three divergent mitochondrial lineages, suggestive of at least three glacial refugia (Skog et al. 2009).

Glacial cycles also have strong impacts on marine ecosystems, and the phylogeography of species within them (Hewitt 2000). Pleistocene glaciations resulted in the lowering of global sea-level by approximately 120 m (Dlabola et al. 2015; Peltier and Fairbanks 2006), which altered coastal marine habitats, disrupted oceanographic currents, and closed off seaways and straits (Bowen et al. 2016). These processes have had pronounced impacts on the connectivity of marine faunal populations, with distinct mitochondrial lineages in the four-eyed sleeper fish *Bostrychus sinensis* (Ding et al. 2018; Qiu et al. 2016), Kentish plover *Charadrius alexandrines* and white-faced plover *C. dealbatus* (Wang et al. 2019), directly attributable to reductions in sea levels and the formation of land bridges between Taiwan and China. Moreover, sea-level change in the Qiongzhou Strait during glacial periods has been linked to population divergence in small-head hairtail *Lepturacanthus savala* (Gu et al. 2021), lipped periwinkle *Monodonta labio* (Zhao et al. 2017), lacustrine goby *Gobiopterus lacustris* (Wang et al. 2018), and silver pomfret *Pampus argenteus* (Sun et al. 2019).

**Fig. 1:**
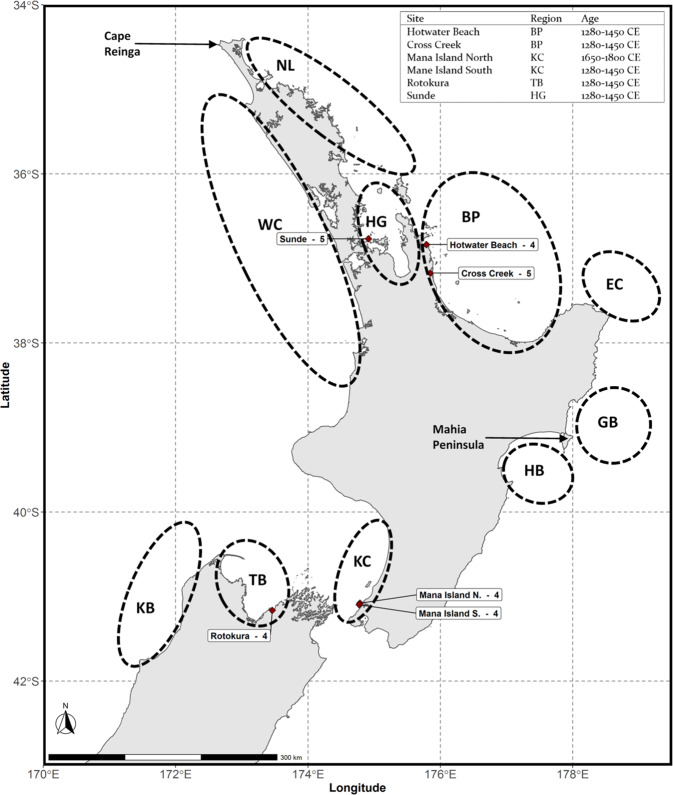
Map showing the sampling distributions of both modern and ancient samples of *C. auratus*. Red diamonds indicate sites where ancient mitochondrial genomes were obtained, numbers behind site names indicate number of successfully sequenced mitochondrial genomes. See Fig. S1 for a map showing all ancient sampling sites and Table S1 for location information. The table on the top right shows the estimated age of the ancient sampling sites, and samples regions to which they are allocated.

Australasian snapper (*Chrysophrys auratus*) is a common coastal fish species in the waters around the North Island, and the northern South Island of New Zealand (Fig. 1). The species also occurs in Australian waters, but shows clear phylogenetic separation suggesting absence of gene flow between the two regions (Tabata and Taniguchi 2000). *Chrysophrys auratus* inhabits waters down to depths of 50 m (max 200 m), and its distribution is primarily determined by water temperature (Parsons et al. 2014), which has a large impact on their growth and survival rate (Wellenreuther et al. 2019). It is predicted that climate change will lead to range expansions as water temperature increase (Brooks 2019). However, little is known regarding their response to historic changes in global climate. Glacial cycles likely had a significant impact on *C. auratus* as reductions in sea levels and ocean temperatures during glacial periods would have affected their suitable habitat (Dlabola et al. 2015; Peltier and Fairbanks 2006). Such environmental changes combined with New Zealand’s isolated geographic location, and its shores running close the edge of the continental shelf, would have led to range contractions and reductions in population size. Phylogeographic signatures of such demographic events will likely be detectable in the contemporary populations.

In recent history, extractive fisheries have had a significant impact on fish populations, particularly in instances of prolonged and intense size-selective fishing (Heino et al. 2015). Artisanal fishing for *C. auratus* began approximately 750 years ago when the East Polynesian ancestors of Māori arrived in New Zealand (Leach 2006; Leach and Davidson 2000). Based on the presence of *C. auratus* bones in archaeological midden (i.e. prehistoric rubbish dumps) deposits (Leach 2006; Seersholm et al. 2018; Smith 2013), *C. auratus* numbers appeared to decline over the ~500 year period of Māori occupation of New Zealand prior to European arrival (Leach 2006). This decline may be related to an increase in catch per unit of effort. There was also a period of climate cooling and environmental unpredictability between 1500–1850 AD (Little Ice Age) (Lorrey et al. 2014; Waters et al. 2017), which may have also influenced fish distributions or recruitment patterns, and therefore fishing success. Morphometric data showed that the average size of *C. auratus* dentaries significantly decreased over this time period (Leach and Davidson 2000). These findings suggest that *C. auratus* population sizes fluctuated over the last 500 years, however, it is unknown to what extent environmental changes and the impacts of artisanal fishing drove these changes (Waters et al. 2017).

Since European arrival, it is well-documented that industrial scale harvesting (beginning in the 1920’s) has contributed to large reductions in population size of New Zealand *C. auratus*, with wild fishery biomass dropping below the soft limit (i.e. 20% of the original biomass) (Fisheries New Zealand 2018). Stocks currently appear to be recovering but it is unclear whether large reductions in abundance caused by intense fishing effort also led to a loss of genetic variation. Previous genetic studies on *C. auratus* in New Zealand utilising nuclear microsatellite loci, reported local losses of genetic variation in a single population with a small effective population size (Hauser et al. 2002).

Recent studies have shown great potential for the retrieval of ancient DNA from fish bones (Ferrari et al. 2021; Oosting et al. 2019), allowing for direct inference of changes in genetic diversity and phylogeographic structure through time. Here we analyse mitochondrial genomes obtained from modern and ancient individuals to assess the phylogeographic structure and demographic history of *C. auratus* throughout New Zealand. Specifically, the following questions are addressed: (1) is there evidence of phylogeographic structure and demographic change associated with Pleistocene glacial cycles; and (2) can past changes in abundance be detected in patterns of genetic diversity, including a potential loss of genetic diversity caused by high fishing pressure since humans colonized New Zealand?

## Methods

### Collection modern samples, extraction and sequencing

Fin clips were collected from 350 modern specimens, obtained from commercial and recreational fishing sources (Fig. 1, Table S1). DNA was extracted using a high-salt extraction protocol adapted from Aljanabi and Martinez (1997), as described in Oosting et al. (2020). Library preparation and high-throughput sequencing were performed by Australian Genome Research Facility (Melbourne, Australia). Double-stranded library preparation was conducted using the Nextera flex protocol with unique dual indexing. High-throughput sequencing was performed on an Illumina NovaSeq 6000 S4.

### Collection ancient samples, extraction and sequencing

Dentaries from ancient *C. auratus* were loaned from the Museum of New Zealand Te Papa Tongarewa, Wellington (Fig. S1). We sampled 124 dentaries from 18 pre-European Māori archaeological middens (Fig. S2 and Table S1). To avoid sampling the same individual multiple times, only left or right dentaries were sampled from each site. Middens were classified as either early (1280–1450 CE) or late (1650–1800 CE) based on what is known regarding Māori subsistence practices during those time periods (Fig. S2) (Smith and James-Lee 2010).

Pre-processing and DNA extraction of ancient dentaries were performed in a dedicated ancient DNA facility (Otago Palaeogenetics Laboratory, Department of Zoology, University of Otago, New Zealand). Portions of the tooth-row were removed in a sterile extraction hood using a Dremel cutting disk (whilst preserving morphometric landmarks) and powdered using a sterile mortar and pestle. Ancient DNA was extracted following Boessenkool et al. (2017), in accordance with standard aDNA protocols (Cooper and Poinar 2000; Knapp et al. 2012), including the use of negative extraction controls.

Library preparation and high-throughput sequencing were performed in a dedicated ancient DNA facility at the University of Oslo (Norway). Double-stranded, double-indexed sequencing libraries were constructed from USER-treated DNA template (Kircher 2012; Meyer and Kircher 2010). Quality of the individual libraries was assessed using an Agilent 5200 fragment analyser using the high sensitivity (HS) assay (DNF-473), following manufacturer’s instructions. Libraries were pooled with the final concentration above 2.0 ng/µl, and sequenced on an Illumina Hi-Seq4000. Sequences were demultiplexed allowing for zero mismatches in the index, yielding approximately 10 million reads per sample (skim sequencing). Endogenous DNA content was determined by mapping sequenced reads to the nuclear genome of *C. auratus* (Catanach et al. 2019). This information was used to identify suitable samples for additional sequencing. Library concentrations for selected samples were adjusted to generate the desired number of reads that allowed the retrieval of mitochondrial genomes with an average coverage of five.

### Read alignment

Sequenced reads from modern and ancient samples were processed using the bam_pipeline in PALEOMIX v1.2.13.3 (Schubert et al. 2014). First, adapter removal v2.2.3 was used to remove the Illumina adapters; using a mismatch rate of 33% (--mm 3) and a minimum read length of 25 bp (--minlength 25). Ambiguous nucleotides (N’s) and low-quality bases were trimmed from 5’ and 3’ read ends. Overlapping paired-end reads from ancient libraries were collapsed into a single read. Burrows-Wheeler Aligner (BWA) v0.7.15 (bwa-mem algorithm) was used to align reads. Reads were simultaneously aligned to the nuclear and mitochondrial genome to minimize false alignment (Ashton 2013; Catanach et al. 2019), creating separate bam files. Unmapped reads and reads with a mapping quality of <25 were removed. PCR duplicates generated in amplification steps during library construction were removed using MarkDuplicates, Picard tools v2.18.20 (Broad Institute 2020). Finally, local realignment was performed using the indel realigner in GATK v4.0.8.1 (McKenna et al. 2010). MapDamage v2.0.9 was used to assess post-mortem DNA damage patterns (elevated number of C-T and A-G misincorporations) to authenticate ancient DNA. (Jonsson et al. 2013). Finally, all hard and soft-clipped reads were removed from BAM files, and indexed using Samtools v1.9.

### SNP calling

Variant calling was performed using GATK v4.1.4.1. First, genomic Variant Call Format (g.vcf) files were generated from the BAM files. This was done using GATK’s HaplotypeCaller, using the mitochondrial genome of *C. auratus* as a reference (Ashton 2013), setting ploidy to 1 (-ploidy 1) and the reference confidence mode set the gvcf (-ERC GVCF). A g.vcf file was generated for each individual and used for joined genotyping. G.vcf files were subsequently merged using GATK’s CombineGVCFs function (default settings). This deviated from GATK’s best practices and was chosen because that function allows the user to specify the ploidy of the sample. Genotypes from both modern and ancient samples were jointly called using GenotypeGVCFs using the following settings: -G StandardAnnotation; -ploidy 1; -new-qual. The vcf file was filtered using bcftools v1.9, and vcftools v0.1.16. In bcftools sites we filtered on possible strand bias (FS < 60.0, SOR < 4), mapping quality (MQ > 30), and quality by depth (QD > 2.0). In vcftools, sites were filtered on minimum genotype quality (--minGQ 15) and minimum sequencing depth (--minDP 3). FASTA sequences were generated for each individual using GATK’s FastaAlternateReferenceMaker and merged into a single alignment.

### Generating mitochondrial sequences of the Pagrus major *(outgroup)*

Ten whole-genome-sequences from wild-caught red seabream (*Pagrus major*) from Japan were used as the outgroup sequences (Nam et al. 2019). Similar to the alignment *C. auratus* reads, high-throughput sequencing data was aligned to both the nuclear (Shin et al. 2018), and mitochondrial genomes from the same species (Miya et al. 2001). FASTA sequences were visually aligned to those of *C. auratus*. One indel and three invariant sites on the end of the sequences were removed so all sequences were the same length (16,724 base pairs).

### Partitioning and substitution model selection

The mitochondrial genome was annotated in Geneious v11.0.3 by searching for homologous sequences (https://www.geneious.com). The annotated mitochondrial genome of *C. auratus* was compared for consistency to the annotation of the red seabream, obtained from the Mitochondrial Genome Database of Fish, MitoFish (Sato et al. 2018). The mitochondrial genome was split into the individual protein-coding genes, 12 S rRNA, 16 S rRNA, control region (or D-loop), and the 22 tRNA sequences. Intergenic regions were added to the tRNA sequences. Protein coding genes were subsequently partitioned into the 1^st^, 2^nd^, and 3^rd^ codon positions, and concatenated to create 1^st^, 2^nd^, and 3^rd^ codon alignments. Genes with reversed reading frames (from ‘3 to ‘5, e.g. ND6) were grouped as if the 3^rd^ codon was the first. PartitionFinder2 identified four distinct partitions that followed different substitution models (Table S2) (Lanfear et al. 2016), based on the mitochondrial annotation (Fig. S3). First codon positions were grouped with ribosomal and transfer-RNA sequences, while 2^nd^ codon, 3^rd^ codon and D-loop had their own partition model. Substitution models for each partition were estimated using Jmodeltest2 (Darriba et al. 2012). Model selection was evaluated using information criteria (i.e. AIC, aAIC, and BIC), giving the most weight to BIC (Table S3).

### Mitochondrial genome variation

The number of unique haplotypes, number of polymorphic sites, number of sites with missing nucleotides, nucleotide diversity (*π*), haplotype diversity (*h*), genetic diversity (*θ*), were estimated using the R-package pegas (Paradis 2010). Tajima’s D was estimated using DNAsp (Rozas et al. 2017). Tests for significant differences in nucleotide diversity between sampling locations and sample types (modern vs ancient) were performed using a custom script published by Alexander et al. (2016) using 10,000 permutations. An hierarchical AMOVA (1000 permutations) was performed using the R-package apex (Jombart et al. 2017), to test for genetic structure.

### Phylogenetic and demographic history

A maximum-likelihood tree was estimated in IQTREE (Nguyen et al. 2015), using default parameters and 1,000 bootstrap replicates. The consensus tree was used to produce a haplotype genealogy in Fitchi (Matschiner 2016). Fitchi was run using default parameters, except the aesthetics value for node size (-m 0.3) and specifying the ploidy (--haploid). The output of Fitchi was used to group samples into clades. We tested for changes in the relative haplotype abundance between different clades to determine whether certain clades had a potential selective advantage over the past 750 years. Fisher’s exact test was performed to test for significant changes in haplotype distribution over time. To ensure unbiased comparison, the modern data set was randomly subsampled down to the same number of observations as the ancient DNA data set (i.e. 26). Subsampling was bootstrapped 1,000 times to obtain confidence intervals. Calculations were performed using a custom Rscript (R Core Team 2013).

Bayesian analyses were performed using BEAST2 v2.6.2 (Bouckaert et al. 2014). Data partitioning and nucleotide substitution models were based on the results obtained from PartitionFinder2 and Jmodeltest2 (Table S3). A strict clock rate was used for all partitions. Different clock rates were used for the control region and the remainder of the mitochondrial genome, 5.0e^−8^ per site per year (site^−1^/year^−1^), and 3.28e^−9^ site^−1^/year^−1^, respectively (Bowen et al. 2006; Gillooly et al. 2005). The latter estimate was based on the average clock rate of nine fish species presented in Gillooly et al. (2005). The substitution rate was allowed to vary between Codon1+RNA, Codon2, and Codon3 partitions (Fig. S4). Base frequencies were estimated empirically from the data. The phylogeny of *C. auratus* was estimated by applying the Coalescent Exponential Population prior. A second phylogeny was estimated using the Yule prior to assess the divergence time between *C. auratus* and *P. major*. Finally, changes in population size of *C. auratus* through time were assessed using Extended Bayesian Skyline Plot prior. Chain lengths for each of the analyses varied between 50 and 500 million steps, depending on how long it took for runs to converge. All runs incorporated a 10% burn-in. Trace profiles were visually checked for convergence using Tracer v1.7 (Rambaut et al. 2018), and individual parameters had an effective sample size over 200. Phylogenetic trees estimated in BEAST2 were summarized in TreeAnnotator v2.6.2 (Bouckaert et al. 2014), generating a maximum clade credibility tree. A custom script was used to extract heights (age) for each node, including 95% confidence intervals. Demographic trends based on the Extended Bayesian Skyline Plots were generated and visualised in R.

## Results

### Sequencing and analysis of mitogenomes

Modern mitochondrial genomes of *C. auratus* had an average coverage of 286× (151–1,704×), and *P. major* mitogenomes had an average coverage of 270× (38×–1,131−). We generated and skim-sequenced 62 libraries out of the 90 successfully extracted ancient specimens. For these libraries levels of endogenous DNA ranged between 0.003% and 58% (Fig. S5 & Table S4). Based on this initial screening, we sequenced additional reads for 26 libraries, and obtained 26 ancient mitogenomes with an average coverage of 5.02×–55.7× (Fig. S6). MapDamage profiles of sequences obtained from ancient specimens showed the usual fragmentation patterns and elevated number of C-T and A-G misincorporations at either read-end that are associated with authentic ancient DNA (Fig. S7). The majority (n = 22) of the mitochondrial genomes originated from middens associated with early Māori period (1280–1450), with four mitogenomes from the late Māori period (1450–1800) (Table S4).

### Mitochondrial genome variation

In total, 472 polymorphic sites resulted in 233 unique haplotypes (Table 1). The overall haplotype diversity was high (*h* = 0.972) and ranged between 0.932 and 0.989 among populations. High haplotype diversity was also observed in the rarefaction curve (Fig. S8). Nucleotide diversity (*π* = 0.0042–0.0055) and genetic diversity (θ = 54.47–64.35) of modern sequences was consistent across populations (Table 1). We did not detect significant differences in nucleotide diversity between sampled populations (Table S5). Tajima’s D was positive for the total dataset and showed varied between sample locations (Table 1). Diversity estimates were similar compared to the total data and modern dataset (Table 1), with no significant difference in nucleotide diversity between modern and ancient sequences (*p* = 0.343).

**Table 1:** Information for 10 sampled populations, both modern and ancient samples.

Sample Locations	Code	N	Np	Nh	Nm	π	h	θ	T_D_
Northland	NL	50	244	31	25	0.0052	0.942	54.47	2.079*
Hauraki Gulf	HG	40	247	33	21	0.0042	0.978	58.07	0.768
		(5)	(214)	(5)	(88)	(0.0055)	(1)	(84.96)	(1.101)
Bay of Plenty	BP	70	267	51	24	0.0052	0.966	55.41	1.899
		(9)	(183)	(9)	(251)	(0.0043)	(1)	(67.33)	(1.105)
East Cape	EC	20	229	17	22	0.0051	0.979	64.55	1.307
Gisborne	GB	15	206	12	0	0.0055	0.962	63.35	2.011*
Hawke’s Bay	HB	20	222	13	28	0.0054	0.932	62.58	2.023*
West Coast	WC	66	261	54	29	0.0053	0.989	54.84	2.178*
Kapiti Coast	KC	17	209	15	33	0.0049	0.978	61.82	1.587
		(8)	(195)	(6)	(194)	(0.0047)	(0.893)	(75.21)	(1.06)
Tasman Bay	TB	33	226	26	30	0.005	0.975	55.69	2.117*
		(4)	(164)	(4)	(107)	(0.0047)	(1)	(89.45)	(0.703)
Karamea Bight	KB	19	214	16	27	0.0055	0.977	61.23	2.180*
Modern	-	350	466	220	35	0.0051	0.968	72.43	0.425
Ancient	-	(26)	(214)	(18)	(339)	(0.0045)	(0.982)	(56.08)	(2.220*)
total	-	376	472	233	339	0.005	0.972	72.55	0.302

Notes: Translation of abbreviations presented in table, N = number of individuals, Np = number of polymorphic sites, Nh = number of unique haplotypes, Nm = number of sites with missing nucleotides, π = nucleotide diversity, *h* = haplotype diversity, θ = genetic diversity (Waterson), *T*_*D*_ = Tajima’s D. * Indicates a significant *p* value of <0.05. Values in brackets show results from ancient samples.

### Phylogeographic structure and demographic history

**Fig. 2:**
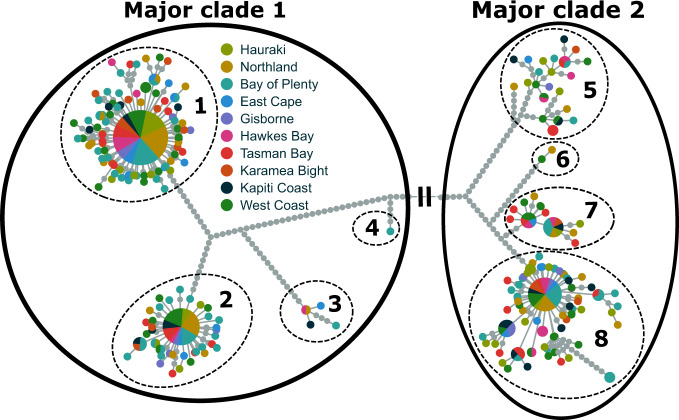
Haplotype genealogy generated in Fitchi, based on maximum likelihood tree produced in IQTREE. Solid black circles show two major clades, and dashed circles show eight minor clades. Colours indicate sample location. Each grey circle represents a single nucleotide change. The black bars indicate a section between the major clades representing 108 nucleotide changes. See Fig. 3 for an unmodified version of the figure that shows the distance between the two major clades.

The haplotype genealogy showed that mitochondrial sequences clustered in two major mitochondrial clades (Figs. 2 and S9), with a sequence divergence (*D*_*A*_) of 0.84%. Eight minor clades were subsequently identified (Fig. 2). The hierarchical AMOVA showed no significant differences between sampled populations (*p* = 0.764). There was also no significant mitochondrial structure observed between two genetic clusters identified using whole-genome data obtained from the high-throughput sequences used for this study (*p* = 0.104) (Northern cluster: Northland, Hauraki, Bay of Plenty, East Cape, Gisborne, and Southern cluster: Hawke’s Bay, Tasman Bay, Karamea Bight, Kapiti Coast, and West Coast) (Fig. S9) (Oosting et al. in prep.). Less than 0.01% of the variation observed between sample locations and nuclear clusters, implying all samples could be treated as single population. There was also no change in the relative abundance of haplotypes between clades over time, *p* = 0.723 and *p* = 0.737 for the major and minor clades respectively.

**Fig. 3:**
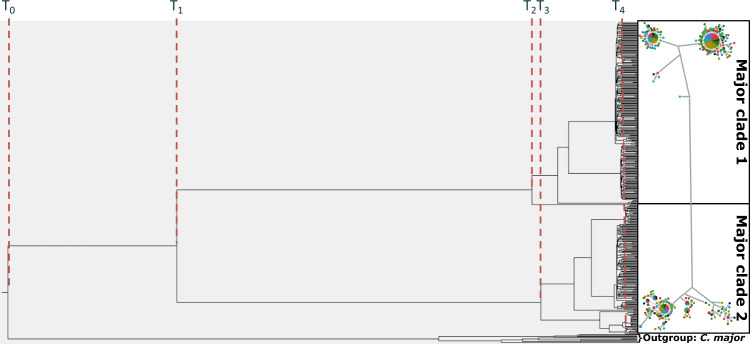
Phylogenetic tree produced with TreeAnnotator. See Table 2 for time estimates for each node. This figure is produced using the Yule prior using modern, ancient, and outgroup samples. Phylogenetic trees generated using the other priors showed the same results, where the older nodes were consistently resolved, and thus shown in a single figure. T_0_ was not estimated for models without an outgroup. The haplotype genealogy is the unmodified version of Fig. 2.

**Fig. 4:**
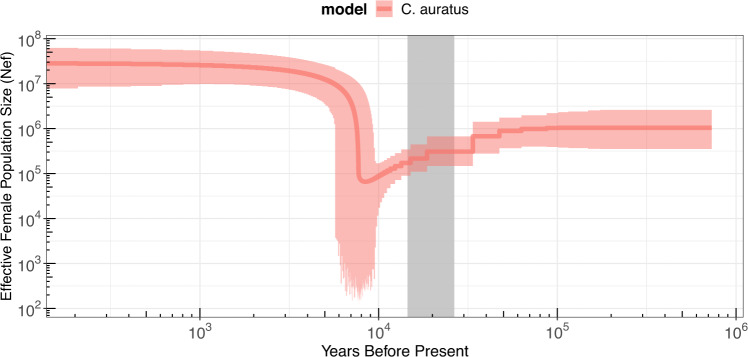
Demographic population trend based on the Extended Bayesian skyline Plot prior using the full dataset (modern and ancient mitochondrial sequences) of *C. auratus*. Solid lines show the mean estimated effective female population size, coloured area shows 95% credible interval. Grey area indicates the approximate timing of the last glacial maximum.

Phylogenetic trees estimated using the Yule and Coalescent Exponential Population models also showed the presence of two highly diverged clades within *C. auratus* (Fig. 3 and Table 2). Branches older than 40,000 years were highly resolved (posterior > 0.99), while younger branches were poorly supported (posterior < 0.5). Based on the Coalescent Exponential Population model, the two major clades separated approximately 650,000 years ago (T_1_) during the mid-Pleistocene (Fig. 3 and Table 2). The Yule model suggested that *C. auratus* and *P. major* (outgroup) diverged around 727,000 years ago (T_0_, 95%CI = 591,000–865,000), which was not significantly different from when the major clades observed in *C. auratus* diverged. Nodes T_2_ and T_3_ were estimated at ~150,000 years ago. A large radiation of new lineages was observed in all models between 7,000 and 35,000 years ago (T_4_) (Fig. 3 and Table 2), consistent with an expansion (Slatkin and Hudson 1991). A population expansion was further supported by the Coalescent Exponential Population model where the growth rate was significantly different from 0 (mean = 3.7238e^−6^, CI95% = 1.3998e^−6^–6.2791e^−6^). The Extended Bayesian Skyline Plot analysis also showed that *C. auratus* experienced an exponential population increase approximately 8,000 years ago (Fig. 4). The analyses estimated that the effective female population size increased by a factor of hundred somewhere after the LGM.

**Table 2:** Node ages estimated using BEAST2.

Data set	Model	Time estimates in x 10,000 years				
		T_0_	T_1_	T_2_	T_3_	T_4_^*^
Modern + Ancient	CEP	–	66.3	16.8	15.9	2.1
			49.0–84.0	11.8–22.2	11.1–20.5	0.8–3.5
Modern + Ancient + outgroup	Yule	72.7	54.8	13.9	13.1	2.6
		59.1–86.5	43.8–66.2	10.3–17.6	9.7–16.6	1.4–3.8

Notes: time estimates are in x 10,000 years. The time estimates indicate the node ages in Fig. 3. Values in italic indicate 95%HPD for each point estimate. *T4 is the point average node age across all nodes younger than 30,000 years. CEP indicates the data set was run using the Coalescent Exponential Population prior, and Yule indicates that the Yule prior was used.

## Discussion

We used contemporary and ancient mitochondrial genomes to investigate the phylogeographic and demographic history of *C. auratus*. Below, we discuss how glacial cycles had a major impact on the phylogeographic structure and population abundance of *C. auratus*. Finally, we will discuss the effect of historic structure on the contemporary estimates of genetic diversity, and the potential effect of exploitation on genetic diversity.

### Phylogeographic structure

The most prominent observation was the presence of two distinct mitochondrial clades among *C. auratus* individuals in New Zealand (Fig. 2). These clades date to a shared common ancestor approximately 650,000 years ago, which is during the mid-Pleistocene (Table 2). The large confidence intervals around the date of the split and uncertainty around clock rates make it difficult to accurately determine the time point of this event (Table 2). It is important to note that that time to the last known common ancestor indicates the maximum divergence time for the two clades. Barriers to gene flow can become established much later than the mutation and divergence process of two lineages, particularly if the ancestral population was large or highly structured populations (Avise 2000). While this phylogenetic split suggests a history of strong population structuring, we did not detect any significant population structure between sample locations, (AMOVA, *p* = 0.764). This two-clade phylogeographic structure typically arises when populations have a significant level of genetic divergence after been in isolation for long periods of time, and have subsequently mixed when barriers to gene flow disappeared (Avise et al. 1987). The subsequent mixing of individuals from both two clades is supported by nuclear data (Oosting et al. in prep.). Glacial cycles throughout the mid-Pleistocene most likely disrupted gene flow with physical barriers to migration (Elderfield et al. 2012). During glacial periods the marine environment would have experienced drastic changes as reductions in sea levels resulted in the formation of land bridges isolating once connected populations (Bowen et al. 2016). This would have had a substantial impact on *C. auratus* populations and gene flow, and was likely an important factor in the formation of the distinct mitochondrial clades (Fig. 2). It is less clear where the geographical separation of these two populations may have occurred. There are various demographic scenarios that could explain the observed ice-age phylogeographic pattern.

Haplotypes from both clades have been detected in Australian samples using mitochondrial control region sequences (unpublished data, Fig. S10), showing trans-Tasman dispersal has occurred. It is possible that the clades diverged on one side of the Tasman Sea and then dispersed to the other side. Both landmasses have coastal environments that have changed significantly as sea level changes formed land bridges over the last 650,000 years, providing the opportunity for population separation and independent lineage formation on either side of the Tasman Sea (Dlabola et al. 2015; Peltier and Fairbanks 2006; Williams et al. 2018). Other possible explanations for the presence of both clades in New Zealand and Australia are more complex, involving repeated bidirectional dispersal across the Tasman Sea. However, we argue that is most likely the two clades were established prior to the arrival of *C. auratus* to New Zealand and Australia, and diverged in the Indo-Pacific region. First, the estimated age of the split between the clades was not significantly different from the 727,000 year old divergence estimate between *C. auratus* and *P. major* (Table 2) (Supplementary information 1), a species that occurs in the South Chinese Sea (Tabata and Taniguchi 2000). The Indo-Pacific is a likely location for where the common ancestor of both species occurred. Second, similar patterns of phylogeographic structure have been observed in other species occurring in the Indo-Pacific region, where glacial cycles reduced sea levels by approximately 120 m, exposing land and thereby separating bodies of water (Bowen et al. 2016; Dlabola et al. 2015; Peltier and Fairbanks 2006). For example, populations of scalloped hammerhead *Sphyrna lewini*, bigscale soldierfish *Myripristis berndti*, and peacock hind *Cephalopholis argus* are thought to have been separated on either side of the Indo-Pacific barrier (what is now Indonesia), after which distinct mitochondrial lineages intermixed during secondary contact (Craig et al. 2007; Duncan et al. 2006; Gaither et al. 2011). Such population fragmentation in the Indo-Pacific ancestral species would pre-date the colonisation and speciation of *C. auratus* in New Zealand and Australia. Overall, this appears to be the most parsimonious explanation for the observed pattern of distinct mitochondrial haplotypes in *C. auratus*.

The haplotype genealogy and phylogenetic trees also showed the presence of eight smaller lineages (minor clades in Fig. 2), which likely diverged over the last 140,000–170,000 years (Fig. 3 and Table 2). Following the same logic as above, these lineages could have diverged through multiple range expansions and contractions throughout the Pleistocene glacial-interglacial cycles as sea levels and temperatures fluctuated over this period (Barrows et al. 2007). Reductions in sea levels and ocean temperatures likely formed isolated pockets of suitable habitat for inshore species such as *C. auratus* (Dlabola et al. 2015). It is possible that these lineages were established when *C. auratus* had already dispersed to New Zealand. Strong phylogeographic structuring has also been observed in New Zealand gastropods (*Cominella* spp), a species with limited dispersal capabilities around New Zealand (Dohner et al. 2018; Fleming et al. 2018). In contrast, species with a broader range of suitable habitats such as tarakihi (*Nemadactylus macropterus*) do not show evidence of phylogeographic structuring (Papa et al. 2021). Range contractions and phylogeographic structuring during the Otiran glacial cycles (74–14 Kya) have also been reported for a wide variety of terrestrial species in New Zealand (Rawlence et al. 2012; Wallis and Trewick 2009). Mitochondrial genomes from Australian samples could be used in the future to corroborate whether these minor clades were established in New Zealand waters. If the minor clades were established in New Zealand, we would expect to see a different phylogeographic pattern in the Australian samples, suggestive of separate demographic histories. It should be noted that while specific physical barriers to gene flow are often used to explain distinct phylogeographic lineages, genetic differentiation in species with large effective population sizes can also arise in populations with either a widespread distribution or limited spawning opportunities (Hogner et al. 2012; Webb et al. 2011).

### Exponential population expansion after the last glacial period

Demographic trends suggest that *C. auratus* went through an exponential population size increase approximately 8000 years ago, which generally coincidences with the Pleistocene-Holocene boundary (Fig. 4) (Walker et al. 2009). The presence of historic structure violates a model assumption of panmixia under which the Extended Bayesian Skyline Plot analyses is performed (Villanea et al. 2020). However, the historic structure has had no significant effect on the analyses as runs performed on each clade separately showed the same demographic trend (Fig. S11). We also confirmed that excluding the ancient sequences from the analyses did not influence the observed patterns in population abundance through time (Fig. S12). Given the uncertainty that can be involved in estimating mutation rates (Ho and Phillips 2009), and the estimating the timing of demographic events (Dos Reis and Yang 2013), the maximum 8000 year-old population increase appears to coincide with a post LGM expansion (29–19 Kya). We hypothesize that the population abundance of *C. auratus* exponentially increased somewhere after the LGM as sea levels rose and the climate warmed (Blunier and Brook 2001; Clark et al. 2009; Newnham et al. 2013; Pahnke et al. 2003). This population expansion was likely facilitated by an increase in suitable habitat, as sea level rise would have extended the available habitat on the continental shelf. *Chrysophrys auratus* most commonly occupy waters up to 50 m, with 200 m considered as their maximum depth (Parsons et al. 2014). Suitable habitat would have been limited and most likely more fragmented during the LGM because the edge of the continental shelf runs relatively close to parts of the New Zealand coast. This can be observed using a rough extrapolation of the suitable habitat using the modern-day bathymetry of New Zealand and lowering the sea level by 120 m (Fig. S13). As sea levels have risen over 120 m in the last 20,000 years (Dlabola et al. 2015; Peltier and Fairbanks 2006), a large proportion of the continental shelf would have become submerged (Fig. S13). Second, the average sea temperature around New Zealand increased by approximately 6 °C (rising from ~10 °C to ~16 °C) after the LGM (Barrows et al. 2007). Increased sea temperature could have facilitated ecological changes (e.g. increased primary production) which may have had a cascading effect through the food web (Cabrera et al. 2022), contributing to the population expansion observed in *C. auratus*. The southern range of contemporary populations of *C. auratus* are primarily dictated by temperature (Parsons et al. 2014). It is logical to assume that the post-LGM increase in ocean temperature would have resulted in a southward expansion of *C. auratus*. With sea surface temperatures expected to rise with 0.8–2.5 degrees in New Zealand by 2100 (Law et al. 2018), *C. auratus* will likely continue to expand their distribution southwards. This idea is supported by ecological niche modelling the distribution of *C. auratus* under different climate scenarios (Brooks 2019), which predicts the species will expand southwards down the West Coast of the South Island under all future climate scenarios.

### Mitogenomic variation

The observed high levels of genetic variation are likely the product of a large effective population size and historic genetic structure. Fish are known to maintain large effective population sizes, which means they can retain high levels of genetic diversity (Ellegren and Galtier 2016; Frankham 1996). This is consistent with exponential population increase *C. auratus* experienced (Fig. 4). High haplotype diversity (*h* = 0.972) and the rarefaction curve show that our sample size was unable to capture most of the mitochondrial diversity present in the population (Table 1) (Fig. S8). Nucleotide diversity (π = 0.005) was very high compared to *Gadus morhua* (π = 0.002) (Martínez-García et al. 2021), a species that has maintained high levels of genetic diversity despite being heavily exploited (Pinsky et al. 2021). This high nucleotide diversity in *C. auratus* had previously been reported based on analyses of mitochondrial DNA control region sequences (Fig. S14) (Papa et al. 2021).

The relatively high nucleotide diversity (π = 0.005) in *C. auratus* can in part be attributed to historic genetic structure. While the mitochondrial clades show no significant genetic structure between contemporary sample locations or clusters based on nuclear loci (AMOVA, *p* = 0.764 and 0.104 respectively), their 650,000-year-old lineages are influencing contemporary diversity estimates. Summary statistics such as nucleotide diversity, genetic diversity and Tajima’s D are estimated using the number of segregating sites. The number of segregating sites is relatively high in *C. auratus* because of the presence of the two highly diverged clades which do not necessarily reflect current day demographics. This may imply that diversity estimates do not correlate well with long-term effective population size (Ellegren and Galtier 2016; Frankham 1996), however, we acknowledge that a number of potentially interacting processes in addition to population size also influence the genetic diversity of wild populations (Bernatchez 2016). For example, the nucleotide diversity in *G. morhua* (π = 0.002) implies that the population is much smaller than that of *C. auratus* (π = 0.005) (Martínez-García et al. 2021). However, estimates of effective female population size in Atlantic cod show similar or larger estimates compared to *C. auratus* (Martínez-García et al. 2021) (Fig. 4). Other mechanisms such as selection or life history traits can influence the relation between diversity and population size (Ellegren and Galtier 2016), however we believe that the historic structure plays a significant role in the comparatively high diversity observed in *C. auratus*. The mitochondrial genome is also a single marker and only captures one gene tree which represents the evolutionary history of the species. The historic structure also effected Tajima’s D, making the interpretation of this summary statistic difficult. This can be observed when comparing the Tajima’s D estimate of the total population with those of the individual clades (Table S6). Here, significant negative Tajima’s D estimates for separate clades suggested a population expansion (*T*_*D*_ = −2.179 & −1.66), which is consistent with the expansion observed in the Extended Bayesian Skyline Plot (Fig. 4).

The ancient mitochondrial genomes provided a direct test for changes in genetic diversity over the last 750 years. First, we did not find evidence for changes in the relative abundance of haplotypes (*p* = 0.723), suggesting there was no recent selective advantage for specific mitochondrial haplotypes. Second, nucleotide diversity has not significantly changed (*p* = 0.349), suggesting no detectable loss of genetic variation since the onset of fishing in New Zealand. This is despite lower temperatures during the Little Ice Age (which could have influenced population abundance) (Lorrey et al. 2014; Waters et al. 2017), the potential impacts of pre-European fishing practices (Leach 2006; Leach and Davidson 2000), and large reductions in population sizes due to industrial-scale harvesting over the last hundred years (Fisheries New Zealand 2018). The ability to detect a genetic signature from a bottleneck depends on the severity, duration, and the amount time since the event (Peery et al. 2012). This would imply that the bottleneck was not severe or long enough for it to effect genetic diversity, or that more time must pass before its effect can be observed in the population. It is also possible that the effects of commercial fishing are not yet detectable in the mitochondrial genome due to low statistical power. Loss of genetic variation was observed in the Tasman Bay using microsatellites (Hauser et al. 2002), yet microsatellites are often selected based on variability (i.e. number of alleles), which implies that heterozygosity estimates can be inflated (Brandström and Ellegren 2008; Väli et al. 2008). Their high variability could make microsatellites more sensitive for detecting reductions in genetic variation. The use of a single marker for this study has limited the power to detect the recent changes in genetic diversity. The addition of nuclear markers will be beneficial in addressing the potential effects of exploitation over the last 750 years. Unfortunately, our approach of shotgun sequencing ancient DNA libraries and low levels of endogenous DNA meant that it was difficult to obtain enough nuclear reads for re-sequencing genomes from past populations. Future efforts may improve this by using capture-based enrichment to extract and genotype nuclear markers to test for changes in genetic variation (Carpenter et al. 2013). Our findings do correspond with similar observations based on nuclear markers in Atlantic cod (*G. morhua*), Atlantic bluefin tuna (*Thunnus thynnus*), and Pacific herring (*Clupea pallasi*), which showed that exploitation has not yet led to detectable losses of genetic diversity (Andrews et al. 2021; Martínez-García et al. 2021; Moss et al. 2016; Pinsky et al. 2021; Speller et al. 2012). Together these studies suggest that heavily exploited species do not appear to have lost any appreciable amounts of genetic diversity. These finding suggest that as stocks are allowed to rebuild, they might have retained much of their pre-exploitation levels of diversity. If correct, this is an encouraging finding for the future of fisheries management. This is also particularly important for resilience as fisheries population adapt to climate change.

## Conclusion

Our findings suggest that Pleistocene glacial cycles have played an important role in the phylogeographic history of *C. auratus*. The end of the last glacial cycle resulted in an exponential population expansion, likely facilitated by increased ocean temperatures and areas of suitable habitat. Current climate change is likely to continue to challenge fisheries species. In some cases, this could result in the expansion of suitable habitat, as is supported by ecological niche modelling of *C. auratus* (Brooks 2019). Finally, we did not find any evidence of a loss of genetic diversity based on data from the mitochondrial genome since the onset of high fishing pressure. Our study has provided new insights into our understanding the phylogeography of a fisheries species and the retention of genetic diversity despite many years of exploitation.

### Data archiving

Mitochondrial genome BAM files have been deposited in the European Nucleotide Archive (PRJEB49332).

## Supplementary information

Supplementary information

Supplementary table S4
