# Supplementary material for: Mitochondrial genomes reveal mid-Pleistocene population divergence, and post-glacial expansion, in Australasian snapper (Chrysophrys auratus)
Source: Heredity (Edinb). 2022 Dec 3;130(1):30–9. doi: 10.1038/s41437-022-00579-1 (PMC9814658; doi:10.1038/s41437-022-00579-1)
Supplement: Supplementary file 1 — Supplementary information [file 41437_2022_579_MOESM1_ESM.pdf]

Supplementary data

Supplementary figures

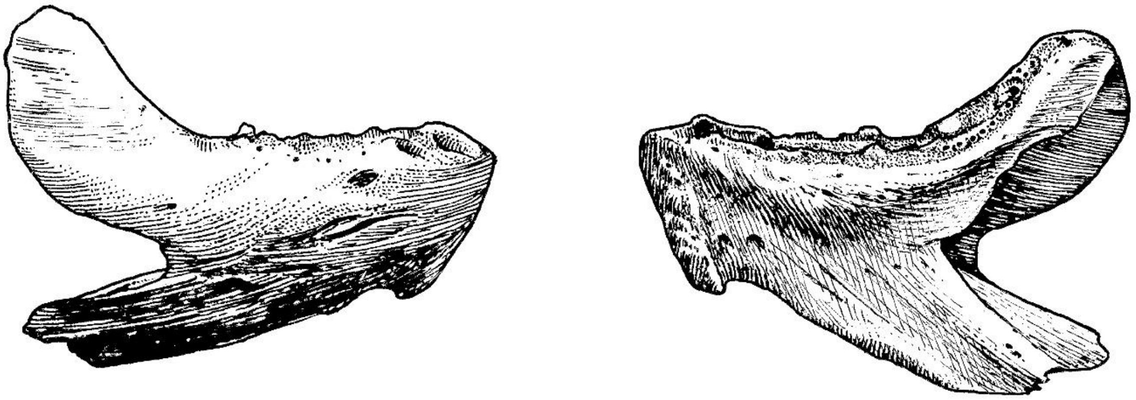

Figure S1: Sketch of left dentary used for identification of ancient *C. auratus* (*Chrysophrys auratus*) samples. *Pagrus auratus* refers to old nomenclature prior the assignment of *C. auratus* for New Zealand and Australian snapper (Parsons *et al*, 2014), source image: Leach (1997).

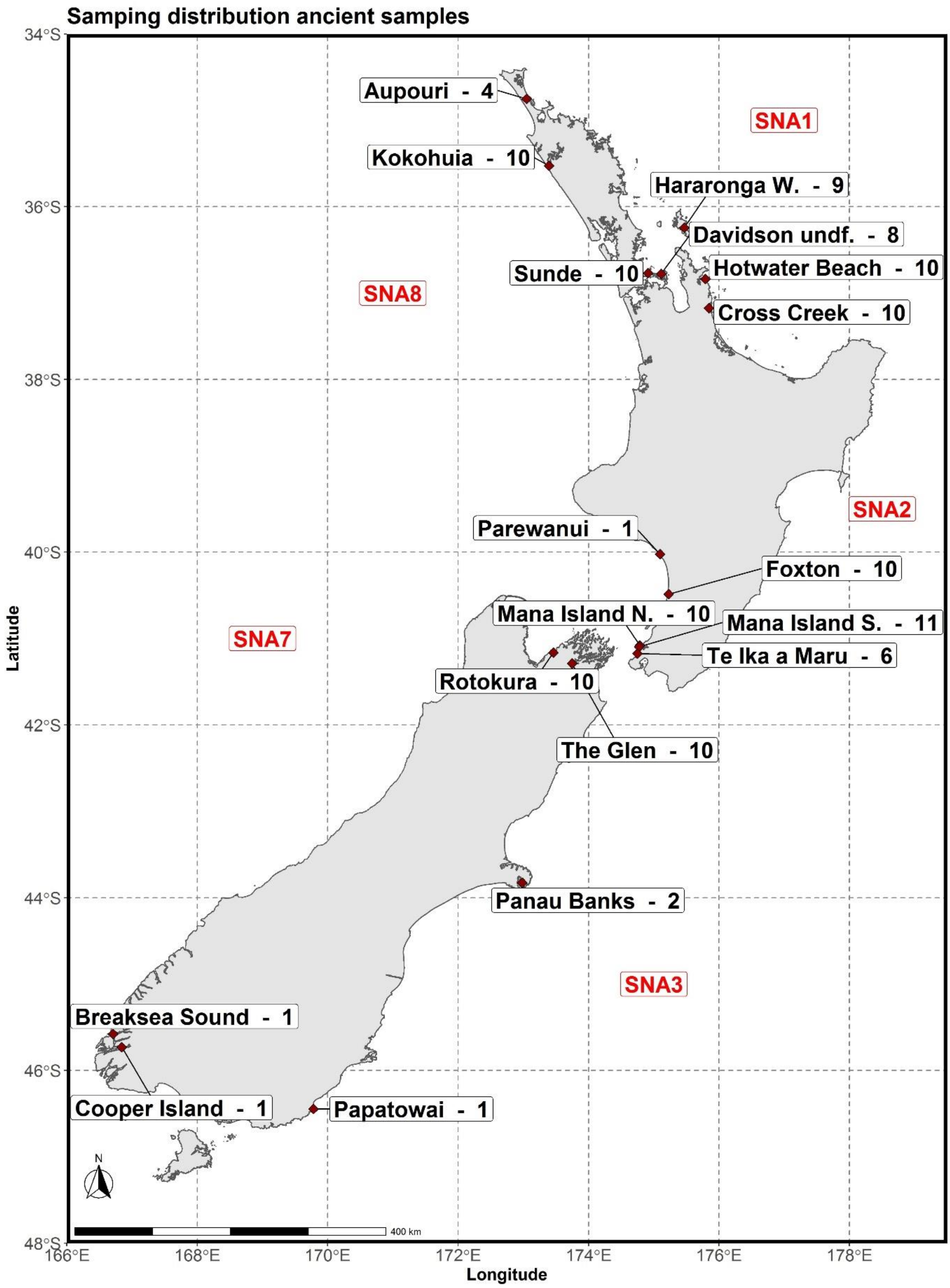

Figure S2: Map showing the sites from which ancient samples were collected. The number behind the site name indicates the number of dentaries collected from that site. Note that not all sites were used for DNA extraction and sequences. For successful sites see Figure 1. SNA# indicate fisheries management areas.

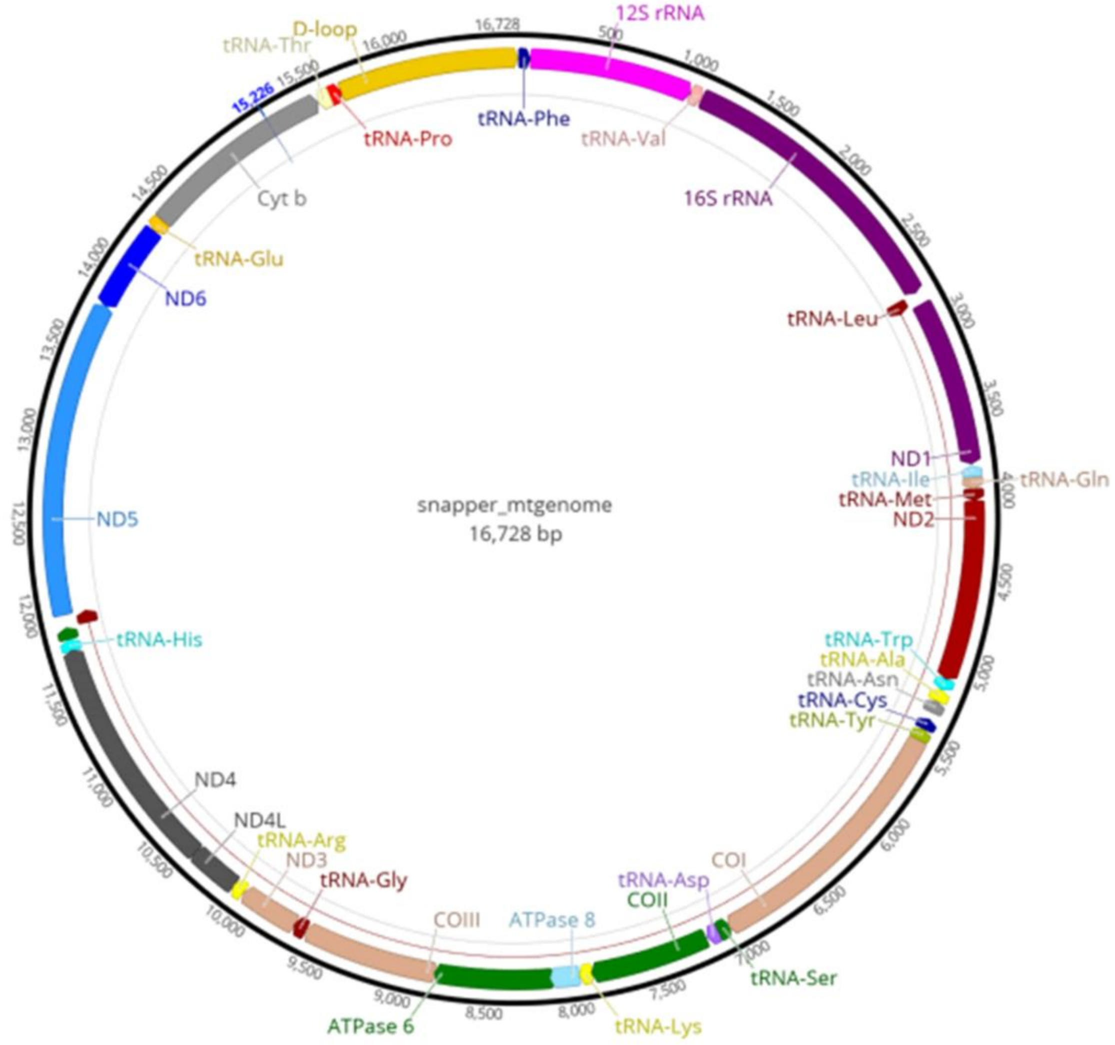

Figure S3: Annotation of *C. auratus* mitochondrial genome, generated in Geneious.

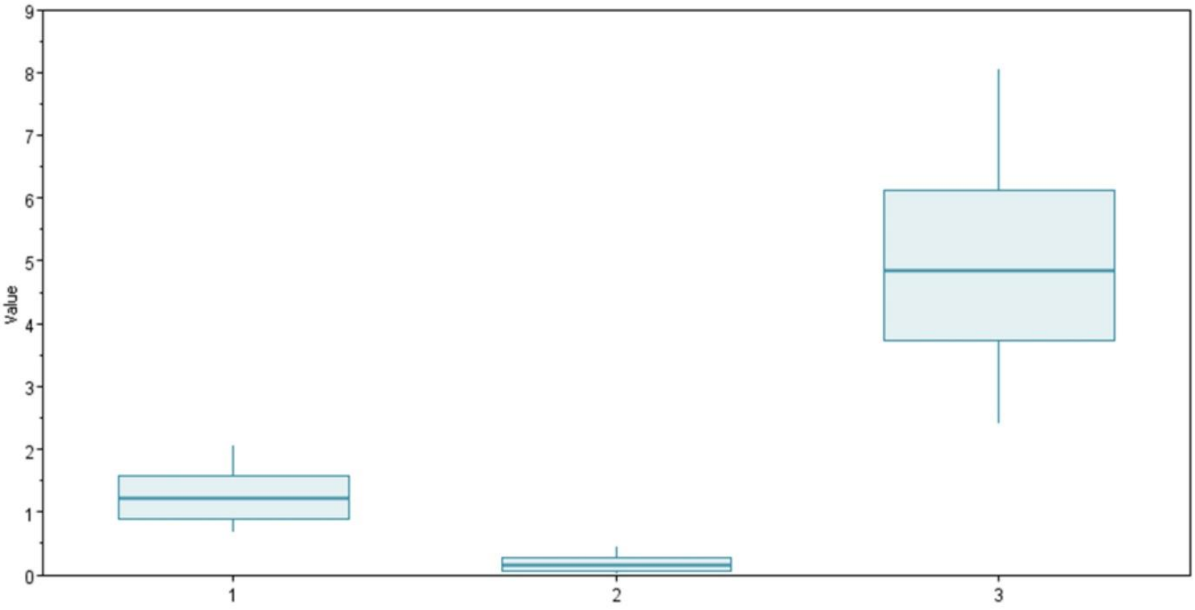

Figure S4: relative substitution rates of the 1st, 2nd and 3rd codons, estimated in BEAST2

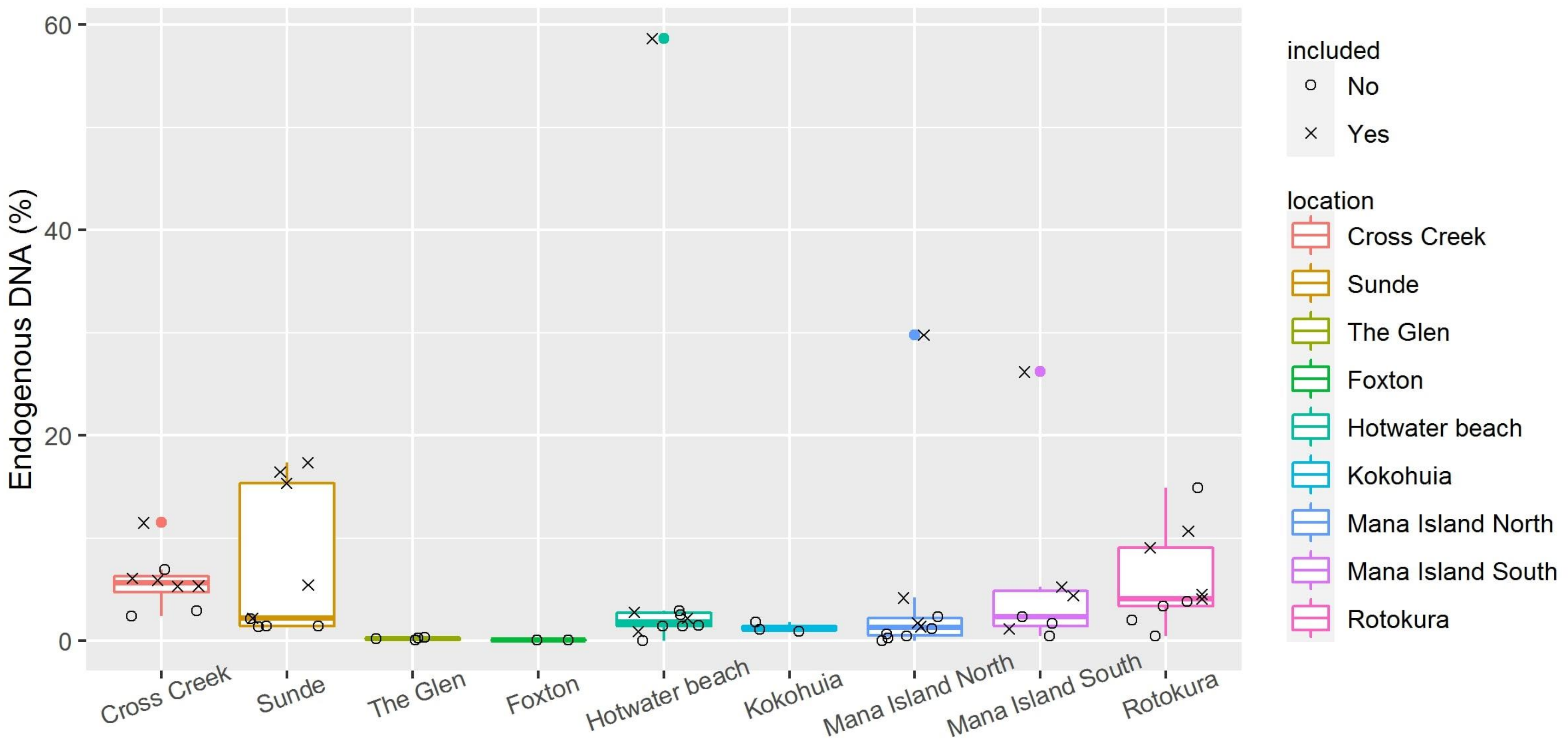

Figure S5: Boxplots showing the levels of endogenous DNA content (%) per site. Black crosses show individual samples that successfully yielded whole mitochondrial genomes. Black circles indicate samples that did not yield whole mitochondrial genomes. Crosses and circles are randomly plotted along the width of the boxplot. The number of samples per area from left to right: 8, 9, 4, 2, 10, 3, 10, 10, 9.

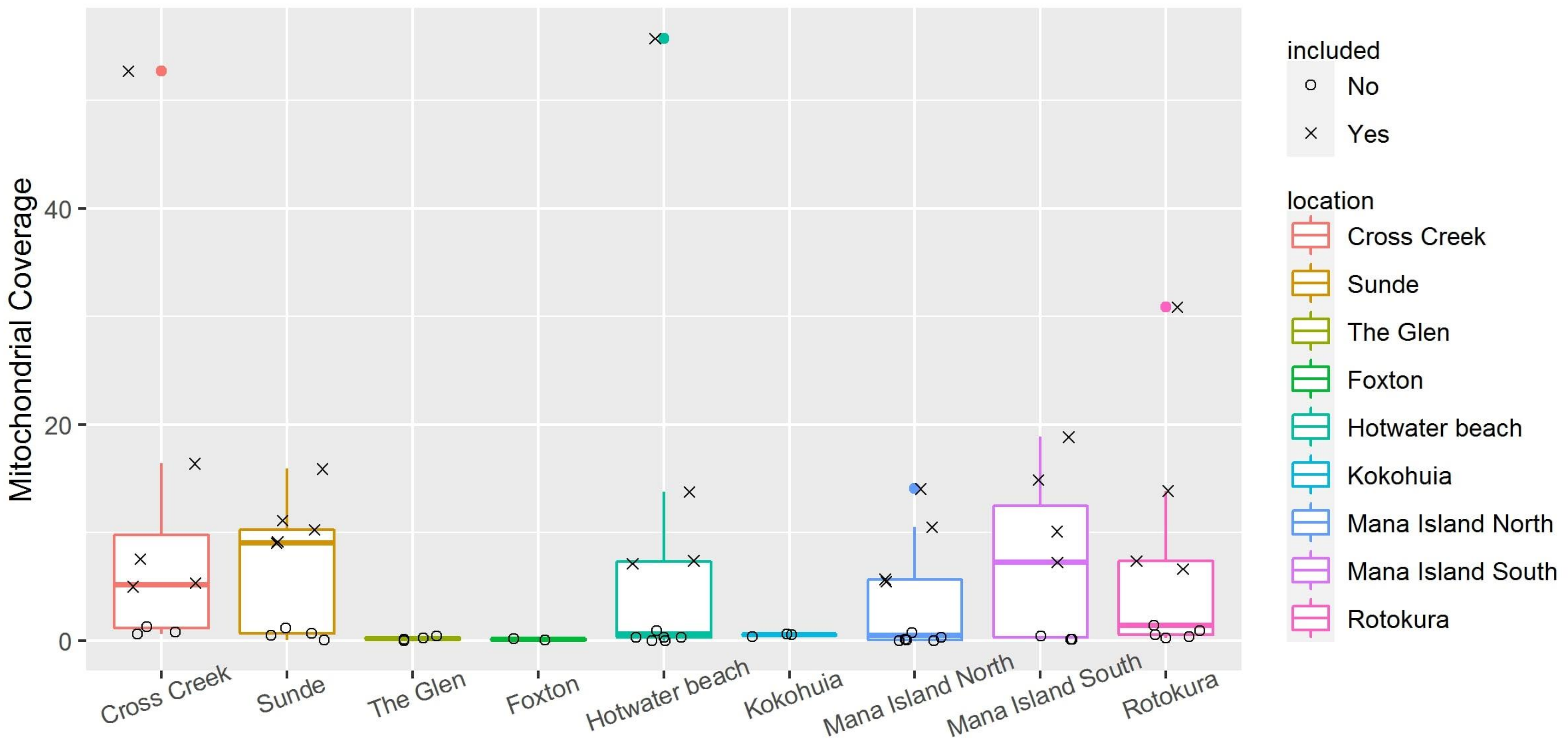

Figure S6: Boxplots showing the mitochondrial coverage of sequenced samples per site. Black crosses show individual samples that successfully yielded whole mitochondrial genomes. Black circles indicate samples that did not yield whole mitochondrial genomes. Crosses and circles are randomly plotted along the width of the boxplot. The number of samples per area from left to right: 8, 9, 4, 2, 10, 3, 10, 10, 9.

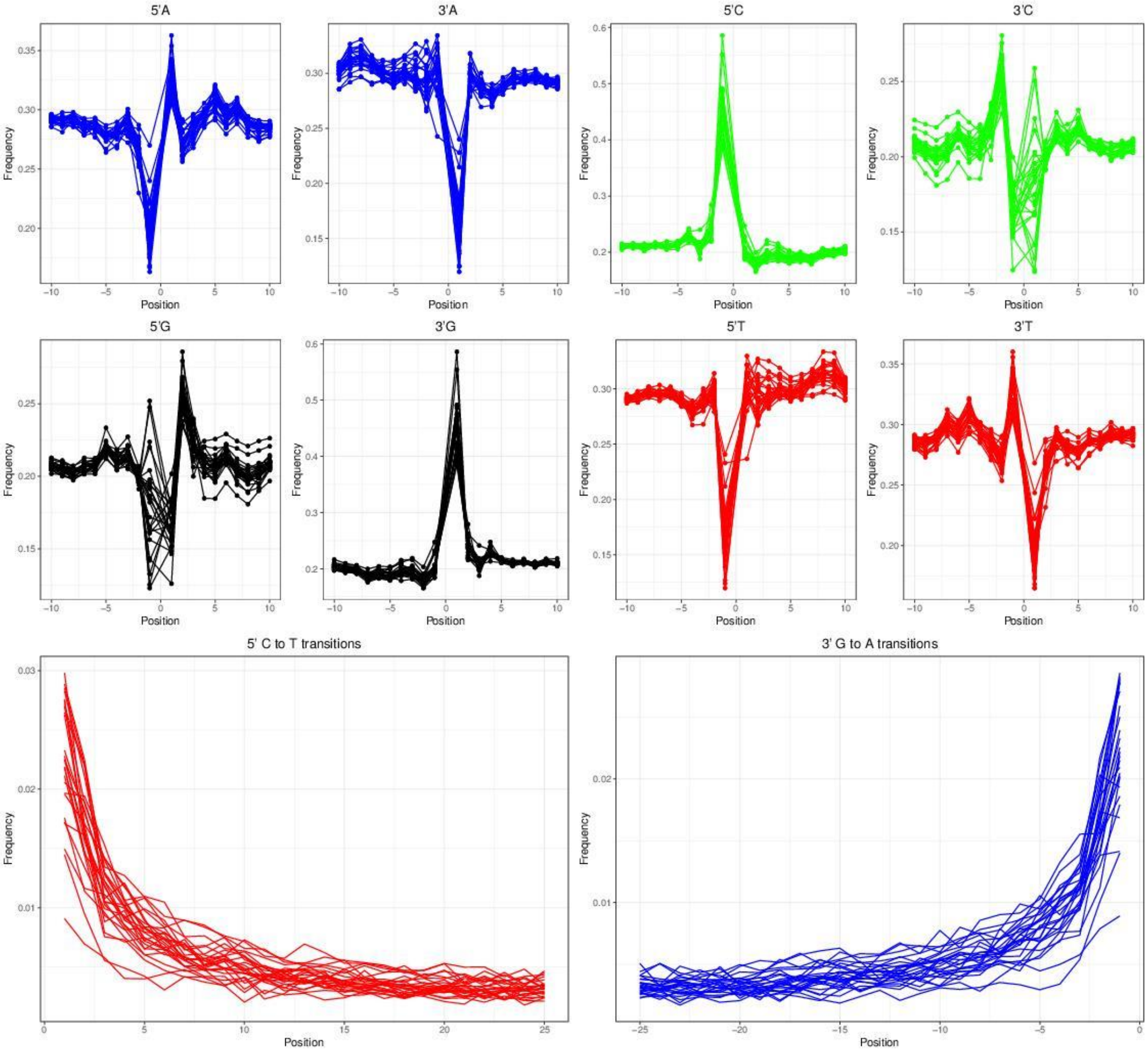

Figure S7: MapDamage results from all ancient samples (n 26) which yielded mitochondrial genomes. Reads were USER-treated resulting in minimal deamination accumulation at read ends. This damage pattern is representative for all samples that yielded complete mitochondrial genomes. Each line represents MapDamage patterns for a single sample.

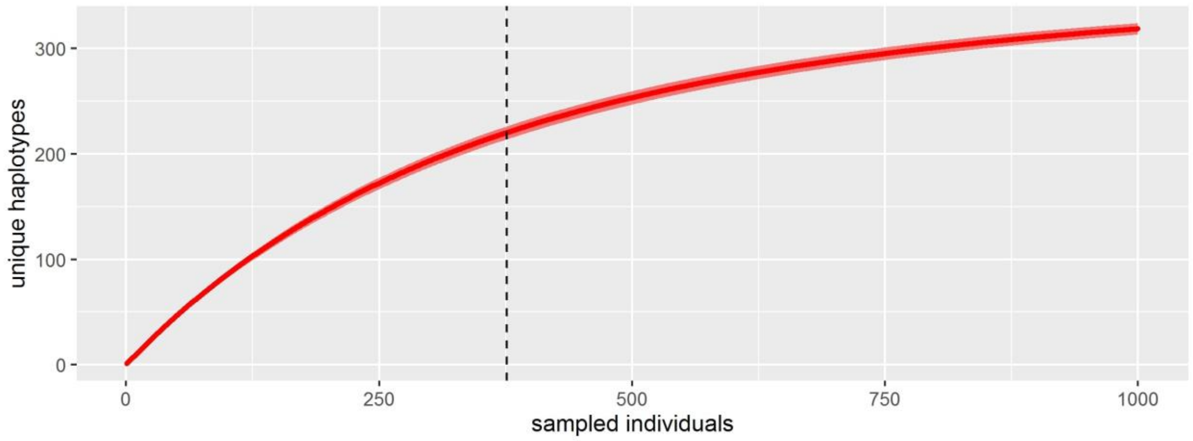

Figure S8: Rarefaction curve of mitochondrial haplotypes.

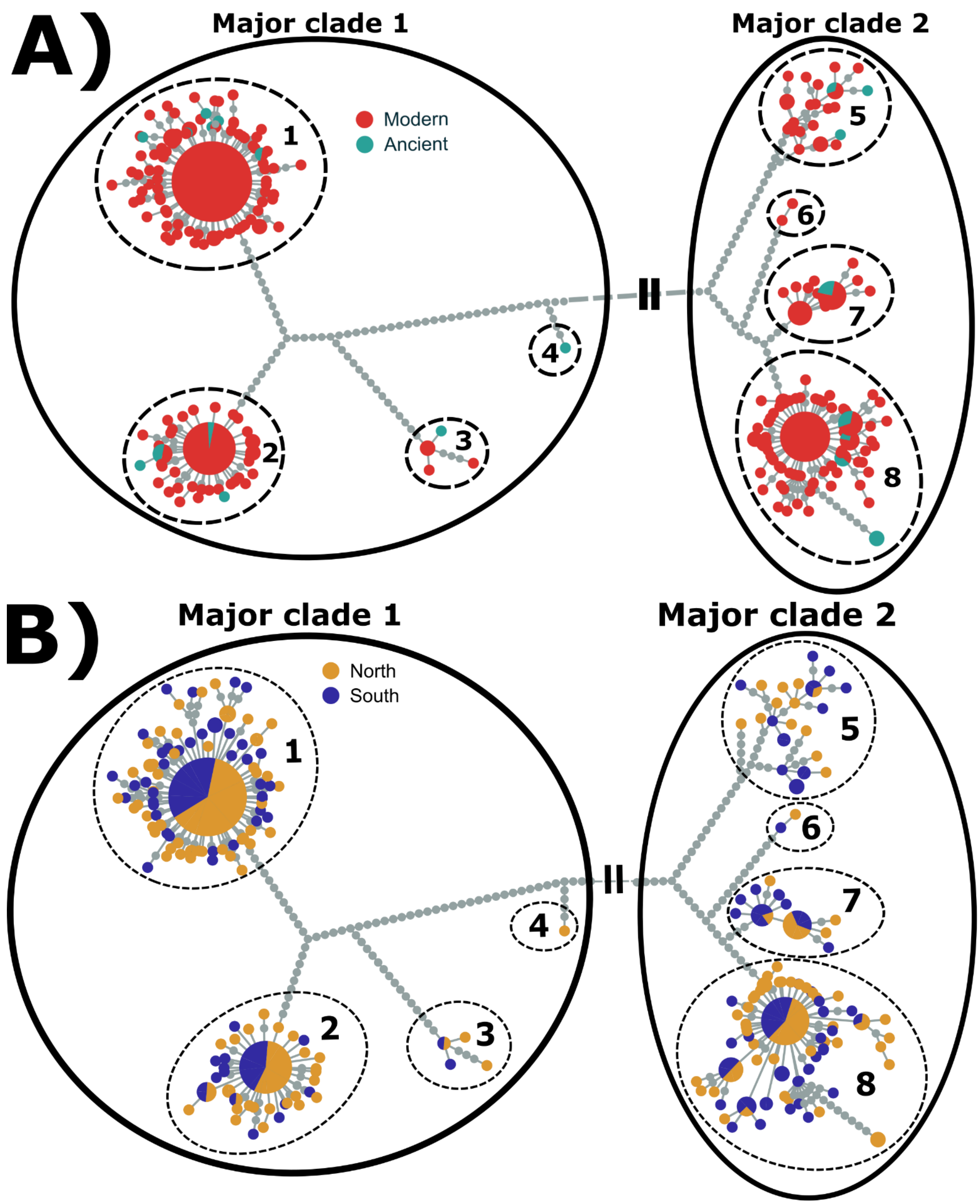

Figure S9: Haplotype genealogy showing *C. auratus* haplotypes based on complete mitochondrial genomes. A) haplotypes colored by samples type. B) haplotypes colored by genetic cluster based on whole-genome data obtained from the high-throughput data used for this study (Oosting et al. in prep.) (see results in main text).

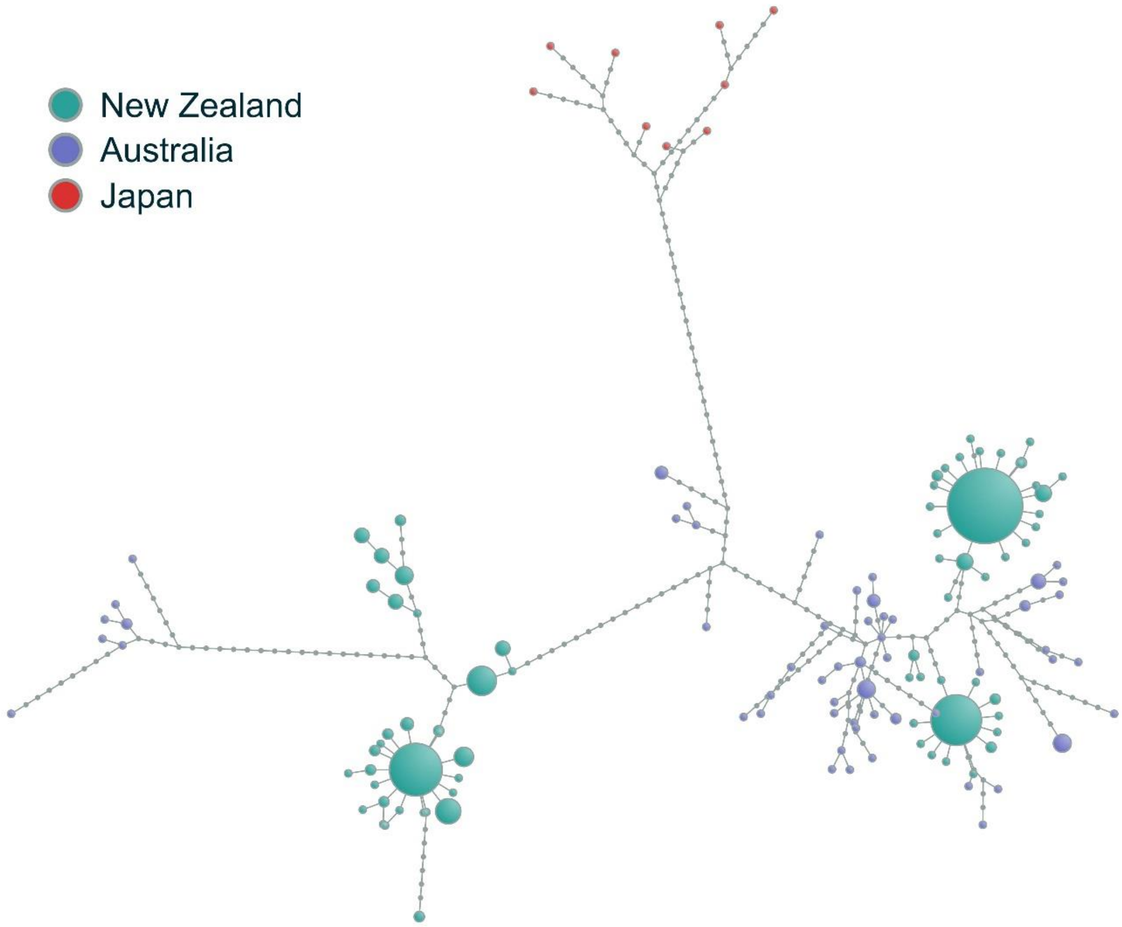

Figure S10: Haplotype genealogy based on the mitochondrial control sequences of *C. auratus* and *P. major*

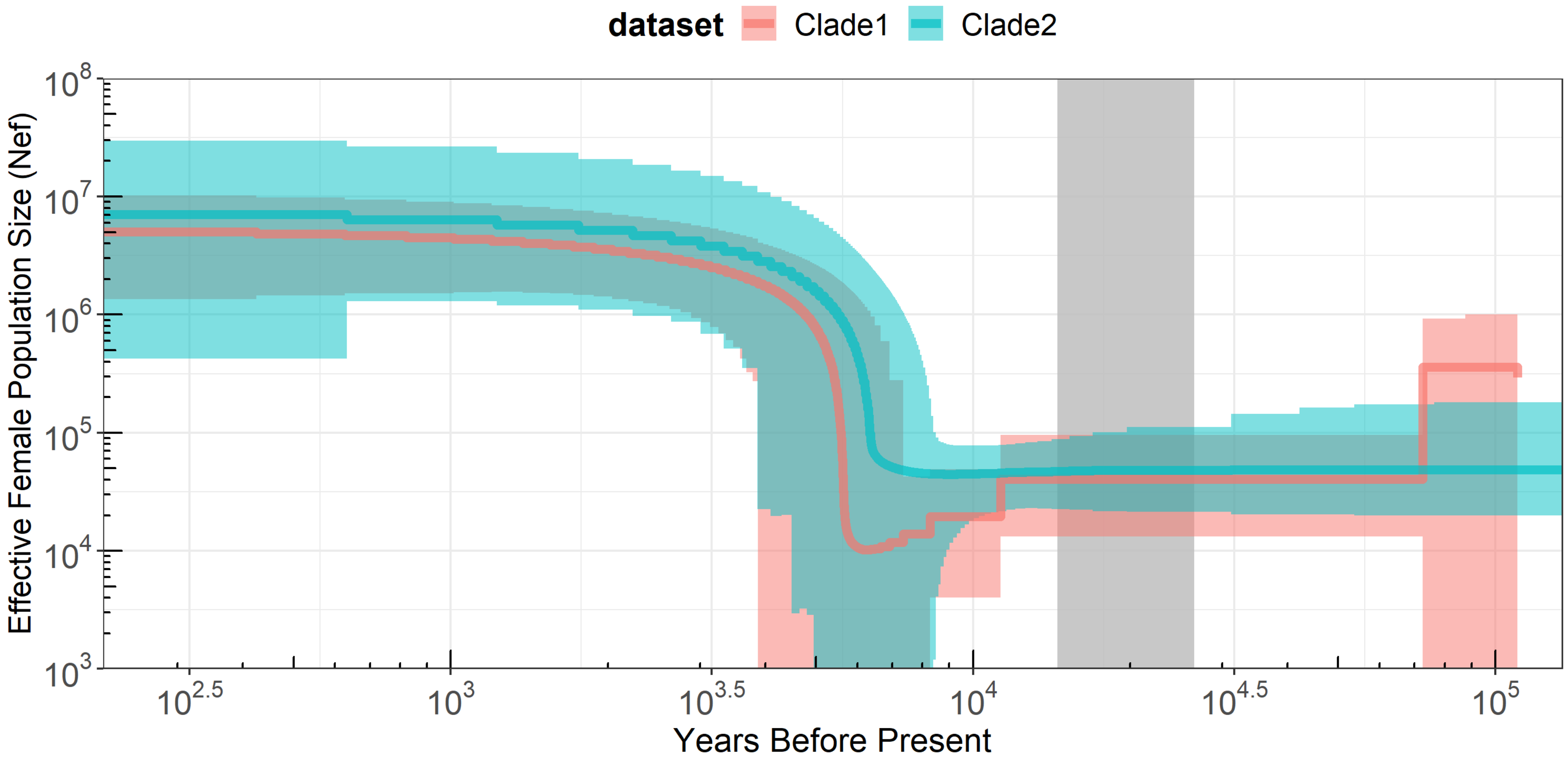

Figure S11: Demographic population trend based on the Extended Bayesian skyline Plot prior shown for both major clades separately (modern and ancient mitochondrial sequences) of C. auratus. Solid lines show the mean estimated effective female population size, coloured area shows 95% credible interval. Grey area indicates the approximate timing of the last glacial maximum.

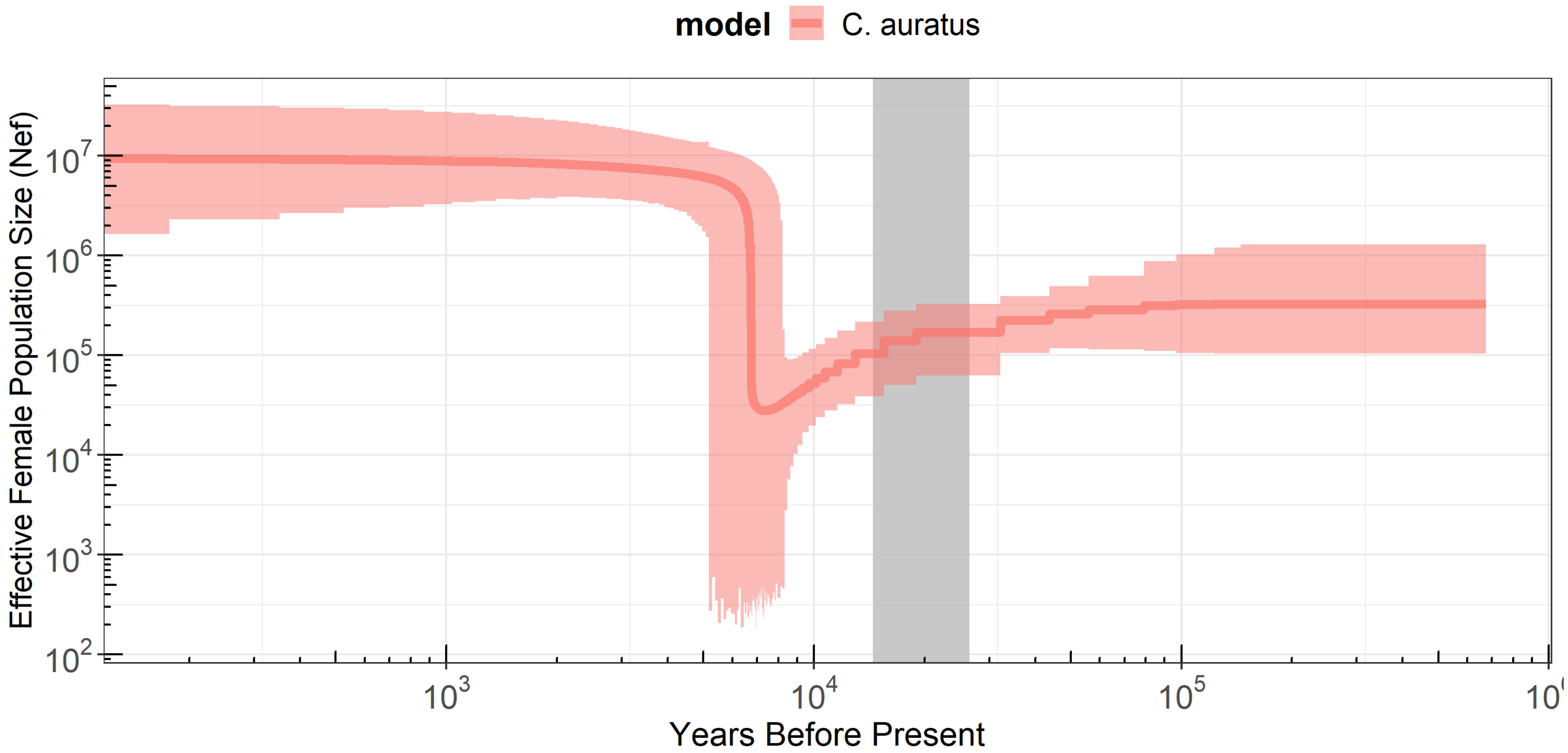

Figure S12: Demographic population trend based on the Extended Bayesian skyline Plot prior using only modern sequences of C. auratus. Solid lines show the mean estimated effective female population size, coloured area shows 95% credible interval. Grey area indicates the approximate timing of the last glacial maximum.

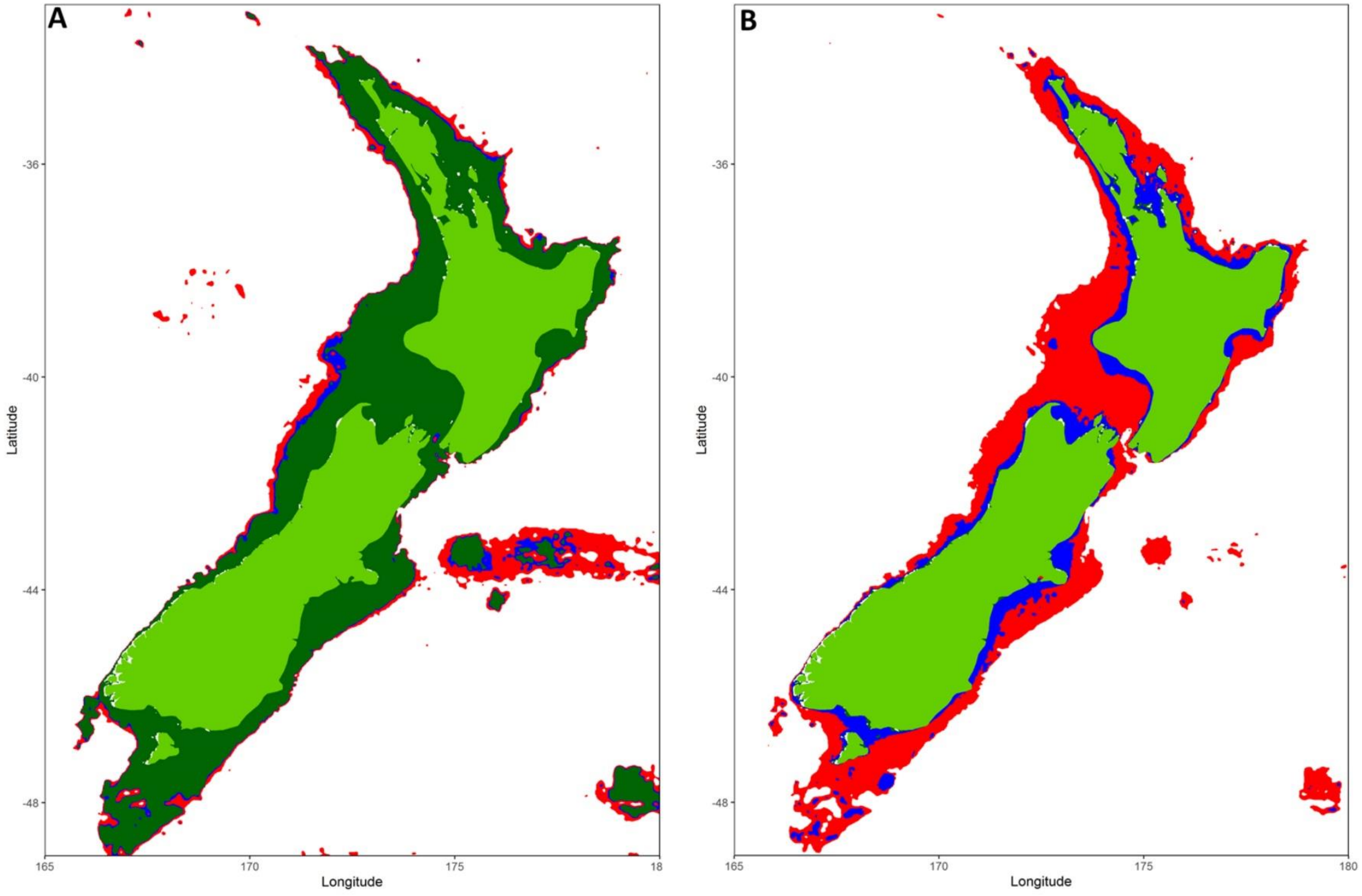

Figure S13: Rough extrapolation of available C. auratus habitat A) during last glacial maximum (LGM) with sea level 120m below current, and B) current available C. auratus habitat. Green shows the current terrestrial surface of New Zealand, dark green additional terrestrial surface that would have been above water during the LGM, blue is C. auratus habitat up to 50m (main distribution), red is C. auratus habitat up to 200m (max depth).

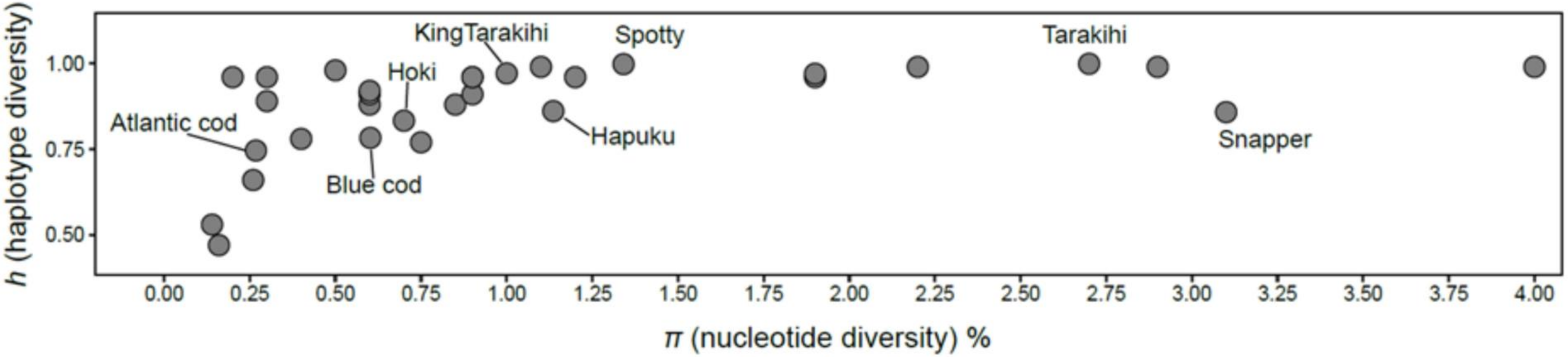

Figure S14: Haplotype (h) and nucleotide (π) diversity estimates of the mtDNA control region in a range of marine fish populations (adapted from Grant and Waples (2000)). Values from the New Zealand species and the Atlantic cod are labelled: Atlantic cod (Gadus morhua), blue cod (Parapercis colias), hāpuku (Polyprion oxygeneios), hoki (Macruronus novaezelandiae), snapper (Chrysophrys auratus), and spotty (Notolabrus celidotus). Source image: Papa et al (2021).

Supplementary tables

Table S1: Sampling information: ancient samples per location

Location	Map name	Period	Sampled	Extracted	Sequenced	Full Mito	Latitude	Longitude
Aupouri	Aupouri	Late	4	0	0	0	-34.751	173.0488
Breaksea Sound	Breaksea Sound	Late	1	0	0	0	-45.577	166.7096
Cooper island	Cooper Island	Early	1	0	0	0	-45.732	166.8417
Cross Creek	Cross Creek	Early	10	8	8	5	-37.174	175.8451
Davidson undefended site	Davidson undf.	Early	8	0	0	0	-36.777	175.1128
Foxton	Foxton	Early	10	2	2	0	-40.486	175.2268
Hararonga West	Hararonga W.	Early	9	0	0	0	-36.244	175.4669
Hotwater Beach	Hotwater Beach	Early	10	10	10	4	-36.837	175.7893
Kokohuia	Kokohuia	Late	10	3	3	0	-35.526	173.3921
Mana Island North	Mana Island N.	Late	10	10	10	4	-41.079	174.7849
Mana Island South	Mana Island S.	Early	11	7	7	4	-41.096	174.7736
Panau Banks	Panau Banks	Late	2	1	1	0	-43.827	172.9834
Papatowai	Papatowai	Early	1	0	0	0	-46.446	169.7813
Parewanui	Parewanui	Late	1	0	0	0	-40.022	175.095
Rotokura	Rotokura	Early	10	8	8	4	-41.376	173.3097
Sunde	Sunde	Early	10	9	9	5	-36.925	174.7265
Te Ika a Maru	Te Ika a Maru	Late	6	0	0	0	-41.174	174.748
The Glen	The Glen	Early	10	2	2	0	-41.288	173.7474
Total	-	-	124	60	60	26	-	-

Notes: Period indicates from what time period each originates, Early = 1280-1450 CE, Late = 1650-1800 CE

Table S2: Results Jomeltest2 – substitution models for each mitochondrial partition

codon1_RNA									
AIC			AICc			BIC			
Model	weight	cum Weight	Model	weight	cum Weight	Model	weight	cum Weight	
GTR+I	0.30402	0.304023	TrN+I	0.26675	0.26675	TrN+I	0.73784	0.73784	
TrN+I	0.18573	0.489753	GTR+I	0.23918	0.50592	TrN+G	0.23316	0.971	TRN+I
GTR+I+G	0.14908	0.638835	TrN+I+G	0.10596	0.61188	TrN+I+G	0.01083	0.98182	
TrN+I+G	0.09014	0.728973	GTR+I+G	0.0959	0.70778	TIM1+I	0.00821	0.99004	
GTR+G	0.08894	0.817917	TrN+G	0.08429	0.79207	TrN	0.00369	0.99373	

codon2									
AIC			AICc			BIC			
Model	weight	cum Weight	Model	weight	cum Weight	Model	weight	cum Weight	
TrN	0.22477	0.224769	HKY	0.28718	0.28718	HKY	0.88716	0.88716	
HKY	0.15271	0.37748	TrN	0.25572	0.5429	TrN	0.05745	0.94461	HKY
TrN+I	0.08274	0.460222	HKY+I	0.06415	0.60705	HKY+I	0.01441	0.95902	
TrN+G	0.0823	0.542522	TPM1uf	0.06392	0.67097	TPM1uf	0.01436	0.97338	
TIM1	0.08214	0.624661	HKY+G	0.06355	0.73452	HKY+G	0.01428	0.98766	

codon3									
AIC			AICc			BIC			
Model	weight	cum Weight	Model	weight	cum Weight	Model	weight	cum Weight	
GTR	0.34653	0.34653	GTR	0.27071	0.27071	TrN	0.92584	0.92584	
GTR+I	0.17676	0.523292	TrN	0.22431	0.49501	TIM1	0.0317	0.95753	TRN
GTR+G	0.16813	0.691426	TIM1	0.10549	0.6005	TrN+I	0.0207	0.97823	
GTR+I+G	0.06468	0.756101	GTR+I	0.08322	0.68371	TrN+G	0.01961	0.99784	
TrN	0.06321	0.81931	GTR+G	0.07915	0.76287	TIM1+I	0.0007	0.99854	

D-loop									
AIC			AICc			BIC			
Model	weight	cum Weight	Model	weight	cum Weight	Model	weight	cum Weight	
GTR+I+G	0.5151	0.515097	HKY+I	0.89048	0.89048	TrN+I+G	0.72066	0.72066	TRN
TrN+I+G	0.24823	0.763329	HKY+I+G	0.06016	0.95064	HKY+I+G	0.11684	0.8375	+ I
TIM1+I+G	0.21257	0.975902	TrN+I	0.04736	0.998	TrN+I	0.09199	0.92949	+ G
GTR+I	0.00736	0.98326	TrN+I+G	0.00116	0.99916	TIM1+I+G	0.05175	0.98123	
TVM+I+G	0.00704	0.990299	TPM1uf+I	0.00082	0.99998	TIM1+I	0.00735	0.98858	

Notes: models indicate mutation models evaluated by Jmodeltest2. weight shows how good each model fits to the observed data. cumWeight indicates the cumalative weight which sums to 1 over all evaluated mutatation models. Only the top 5 mutation models are shown for each partition

Table S3: Substitution models and clock rates for partition used in all BEAST2 analyses

Partition	Clock rate	Sub model	Kappa 1	Kapp2	Inv	G count	G shape
Codon1+RNA*		TN93+I	35.6662	14.5694	0.845	-	-
Codon2	3.28E-09	HKY	1.25	-	-	-	-
Codon3		TN93	77.0797	14.8292	-	-	-
Control region	5.00E-08	TN93+G	59.2829	25.9365	-	4	0.017

Notes: Sub model=substitution model, Inv=proportion of invariant sites, G count=Gamma category Count, G shape = Gamma shape. *RNA indicates mitochondrial sequences coding for the 12s, 16s and tRNA's

Supplementary table S4 was uploaded as a separate file

Table S5: Nucleotide diversity difference between populations below diagonal, p-values above diagonal

	Bay of Plenty	Bay of Plenty ancient	East Cape	Gisborne	Hauraki	Hauraki Ancient	Hawkes Bay	Kapiti Coast	Kapiti Coast ancient	Karamea Bight	Northland	Tasman Bay	Tasman Bay ancient	West Coast
Bay of Plenty	--	0.1536	0.5707	0.2408	0.1594	0.7169	0.1441	0.1637	0.2332	0.4807	0.992	0.2748	0.6008	0.2772
Bay of Plenty ancient	0.00102	--	0.338	0.5857	0.4785	0.8497	0.6221	0.6482	0.8819	0.1338	0.162	0.4064	0.7454	0.3419
East Cape	0.00024	0.00078	--	0.5156	0.5845	0.8465	0.4076	0.4213	0.4521	0.3148	0.5649	0.7478	0.7923	0.8883
Gisborne	0.0006	0.00042	0.00036	--	0.8163	0.9713	0.9079	0.8819	0.7313	0.1827	0.2591	0.6655	0.9651	0.566
Hauraki	0.00047	0.00055	0.00023	0.00013	--	0.9591	0.6661	0.6655	0.6623	0.1361	0.192	0.7724	0.9507	0.5822
Hauraki ancient	0.0007	0.00032	0.00046	0.0001	0.00023	--	0.989	0.9952	0.8954	0.4449	0.7143	0.9294	0.9453	0.923
Hawkes Bay	0.00066	0.00036	0.00041	0.00006	0.00018	4.59E-05	--	0.9632	0.7952	0.1339	0.1637	0.5279	0.9187	0.4149
Kapiti Coast	0.00068	0.00034	0.00044	0.00008	0.00021	2.18E-05	2.41E-05	--	0.8078	0.1471	0.1861	0.5334	0.9014	0.4304
Kapiti Coast ancient	0.00089	0.00013	0.00065	0.00029	0.00042	0.00019	0.00024	0.00021	--	0.1855	0.2475	0.5587	0.8253	0.4913
Karamea Bight	0.00031	0.00133	0.00055	0.00091	0.00078	0.00101	0.00096	0.00099	0.0012	--	0.4895	0.1875	0.5433	0.1828
Northland	0	0.00102	0.00025	0.0006	0.00048	0.0007	0.00066	0.00068	0.0009	0.00031	--	0.3068	0.6124	0.3118
Tasman Bay	0.00038	0.00064	0.00014	0.00022	0.00009	0.00032	0.00028	0.0003	0.00052	0.00069	0.00038	--	0.881	0.8216
Tasman Bay ancient	0.00054	0.00048	0.0003	0.00006	0.00007	0.00016	0.00011	0.00014	0.00035	0.00085	0.00055	0.00017	--	0.8198
West Coast	0.0003	0.00072	0.00006	0.0003	0.00017	0.0004	0.00035	0.00038	0.00059	0.00061	0.00031	0.00007	0.00024	--

Notes: sequences from ancient samples are treated as separate populations (ancient behind sample location) to test for temporal differences in nucleotide diversity per sampling location.

Table S6: Summary statistics given for the total dataset and grouped for each of the identified major clades shown in Figure 2.

dataset	N	Np	Nh	Nm	π	h	θ	T_D
total	376	472	233	339	0.005046	0.972284	72.55428	0.30164
clade1	220	227	131	215	0.00081	0.928684	38.03257	-2.17981**
clade2	156	177	102	248	0.000852	0.98048	31.47303	-1.66321

Notes: Translation of abbreviations presented in table, N = number of individuals, Np = number of polymorphic sites, Nh = number of unique haplotypes, Nm = number of sites with missing nucleotides, π = nucleotide diversity, h = haplotype diversity, θ = genetic diversity (Waterson), TD = Tajima's D. * Indicates a significant p-value of <0.05. Values in brackets show results from ancient samples.

Supplementary information

Supplementary information

Divergence between snapper (C. auratus) and red seabream (P. major)

Methods

Nei's D_A for nucleotide divergence between New Zealand and Japan was estimated using the R package strataG (Archer *et al*, 2017), and based only on the mitochondrial control region sequence. This enabled the divergence rates reported by Tabata and Taniguchi (2000) to be compared.

Results

The mitochondrial control region showed a 4.08% sequences divergence (Nei's D_A) between modern snapper and red seabream (**Error! Reference source not found.**). Combined with clock rate of $5.0e^{-8}$ site^{-1}/year^{-1}, this implies the two species diverged approximately 816 000 year ago. When applying the clock rates used by Tabata and Taniguchi (2000) ($6.0e^{-9}$ & $1.4e^{-8}$ site^{-1}/year^{-1}), the two species diverged between 1.5 and 3.4 million years ago.

Discussion

The closest living relative to snapper is the red seabream which occurs in the Indo-Pacific and is found as far north as Japan. The time to the last known common ancestor between the two species was estimated to have lived around 727 000 years ago (95%CI 591 000-865 000) (Figure 3, & table 2). This estimate corresponds with the divergence time estimate of 816 000 years based on the 4.08% sequence divergence of

the control region and a clock rate 5.0e^{-8}. Similar to the divergence of the two mitochondrial clades in snapper, is it likely that glacial cycles facilitated the divergence between the two species. The current distribution of red seabream includes the Indo-Pacific, an area where glacial cycles are thought to have had a strong influence on phylogeographic structuring and speciation (Bowen *et al*, 2016). After the species had diverged, snapper would have expanded southward to Australia and subsequently New Zealand.

The divergence estimates presented in this study do not correspond with a previous estimate reported by Tabata and Taniguchi (2000). This discrepancy is caused by an error in the interpretation of the sequence divergence (D_A), variation in the estimates and the use of different clock rates. Tabata and Taniguchi (2000) did not consider the fact that their two and six-million-year estimate based on 3.48% sequence divergence (D_A) represents the divergence time of both species. The time when these two species diverged is half their reported estimate, assuming both species diverged at equal rates. The resulting divergence time (one to three million years) still places the divergence time much further back in time. This study also selected a faster clock rate (5.0e^{-8}) compared to the previous study (6.0e^{-9} & 1.4e^{-8}), resulting in a more recent divergence time. Verifying substitution rates is extremely difficult and estimates of divergence time should always be used with caution. The mutation rate used in this study is based on Bowen *et al* (2006), who compared the substitution rates from 10 different fish species. Based on their comparative analyses we argue that a substitution rate of 5.0% per million years is justified. An argument for choosing a faster mutation rate when looking at recent demographic changes is that divergence rates ignore mutations that were not fixed or removed from the population.

References:

Archer FI, Adams PE, Schneiders BB (2017). stratag: An r package for manipulating, summarizing and analysing population genetic data. *Molecular Ecology Resources* **17**(1)**:** 5-11.

Bowen BW, Gaither MR, DiBattista JD, Iacchei M, Andrews KR, Grant WS *et al* (2016). Comparative phylogeography of the ocean planet. *Proc Natl Acad Sci U S A* **113**(29)**:** 7962-7969.

Bowen BW, Muss A, Rocha LA, Grant WS (2006). Shallow mtDNA Coalescence in Atlantic Pygmy Angelfishes (Genus Centropyge) Indicates a Recent Invasion from the Indian Ocean. *Journal of Heredity* **97**(1)**:** 1-12.

Grant WS, Waples RS (2000). Spatial and Temporal Scales of Genetic Variability in Marine and Anadromous Species: Implications for Fisheries Oceanography. In: Harrison PJ and Passons TR (eds) *Fisheries Oceanography An Integrative Approach to Fisheries Ecology and Management*: 2000.

Leach F (1997). *A guide to the identification of fish remains from New Zealand archaeological sites*. Archaeozoology Laboratory, Museum of New Zealand Te Papa Tongarewa.

Papa Y, Halliwell AG, Morrison MA, Wellenreuther M, Ritchie PA (2021). Phylogeographic structure and historical demography of tarakihi (Nemadactylus macropterus) and king tarakihi (Nemadactylus n.sp.) in New Zealand. *New Zealand Journal of Marine and Freshwater Research***:** 1-25.

Parsons D, Sim-Smith C, Cryer M, Francis M, Hartill B, Jones E *et al* (2014). Snapper (Chrysophrys auratus): a review of life history and key vulnerabilities in New Zealand. *New Zealand Journal of Marine and Freshwater Research* **48**(2)**:** 256-283.

Tabata K, Taniguchi N (2000). Differences between Pagrus major and Pagrus auratus through mainly mtDNA control region analysis. *Fisheries Science* **66**(1)**:** 9-18.
